# Exocyst subunits EXO70B1 and B2 contribute to stomatal dynamics and cell wall modifications

**DOI:** 10.3389/fpls.2025.1694769

**Published:** 2025-12-17

**Authors:** Matěj Drs, Karel Müller, Aline Voxeur, Jitka Ortmannová, Natalia Serrano, Samantha Vernhettes, Eliška Škrabálková, Martin Potocký, Viktor Žárský, Tamara Pečenková

**Affiliations:** 1 Laboratory of Cell Biology, Institute of Experimental Botany of the Czech Academy of Sciences, Prague, Czechia; 2 Department of Experimental Plant Biology, Faculty of Science, Charles University, Prague, Czechia; 3 Laboratory of Hormonal Regulations in Plants, Institute of Experimental Botany of the Czech Academy of Sciences, Prague, Czechia; 4 Université Paris-Saclay, Institut National de Recherche pour l'Agriculture, l'Alimentation et l'Environnement (INRAE), AgroParisTech, Institut Jean-Pierre Bourgin for Plant Sciences (IJPB), Versailles, France

**Keywords:** exocyst, stomata, cell wall, gene expression, defense

## Abstract

**Introduction:**

Based on previous reports of defense-related defects in the *Arabidopsis* loss-of-function (LOF) mutants of the EXO70B1 and EXO70B2 exocyst subunits, we investigated the underlying causes of these phenotypes.

**Methods:**

We analyzed stomatal aperture states in both young and adult plants. As the exocyst is involved in the secretion to the cell wall, we examined cell wall composition, and we correlated these findings via a comprehensive mRNA expression analysis.

**Results:**

Our results revealed and discovered a more closed initial state of stomatal opening in *exo70B* mutants, as well as altered methylation and acetylation modifications of pectin and hemicellulose in the studied mutant lines. These changes in cell wall modifications may contribute to both compromised stomatal aperture-dependent and stomatal aperture-independent defense responses, as well as to the transcriptional activation of defense pathways observed in non-infected mutant plants at adult developmental stages. Several candidate genes involved in these processes were pinpointed using RNA-seq analysis.

**Discussion:**

Interestingly, although the primary phenotypic and RNA-seq deviations in young mutant lines may be specific for each of the two EXO70B mutant lines, they exhibit converging gene expression profiles in later developmental stages. This convergence may reflect the shared evolutionary origin of the two Brassicaceae EXO70 isoforms by duplication from a common ancestral gene.

## 1 Introduction

The exocyst complex is an evolutionarily conserved secretory vesicle-tethering hetero-octameric protein complex, first described in yeast and later found throughout kingdoms, that regulates the process of vesicles tethering to the target plasma membrane (Hsu et al., 2004; TerBush et al., 1996). The EXO70 and SEC3 subunits direct the complex to the target membrane through an interaction with membrane phosphatidylinositol phosphate species (Boyd et al., 2004; He et al., 2007; Liu et al., 2007; Pleskot et al., 2015; Synek et al., 2021). In plants, genes encoding exocyst subunits underwent amplification; for instance, the EXO70 subunit in *Arabidopsis thaliana* is represented by eight groups of isoforms (A–H) comprising 23 gene paralogs (Cvrčková et al., 2012; Elias, 2003). It has been found that a variety of plant cellular and physiological functions depend on proper exocyst function—e.g., cytokinesis, polar auxin transport, pollen germination, root hair growth, defense against pathogens, and xylem formation (Cole et al., 2005; Drdová et al., 2013; Fendrych et al., 2010; Hála et al., 2008; Janková Drdová et al., 2019; Kubátová et al., 2019; Kulich et al., 2015; Marković et al., 2020; Ortmannová et al., 2022; Pečenková et al., 2011; Saccomanno et al., 2020; Synek et al., 2006; Vukašinović et al., 2017). Plant exocyst also takes part in the autophagic membrane trafficking, similar to mammalian exocyst (Bodemann et al., 2011; Ji et al., 2020; Kulich et al., 2013). While the EXO70A1 isoform contributes to the tip growth, cytokinesis, and endosomal recycling, the autophagy exocyst function has been mainly assigned to the exocyst complex variants comprising EXO70B isoforms (Acheampong et al., 2020; Brillada et al., 2021; Kulich et al., 2013).

There are two EXO70B isoforms in Brassicaceae, EXO70B1 and EXO70B2, unlike in other plant families, where only one EXO70B is present. It is probable that the B group bifurcation is a consequence of the ongoing arms race type of evolution, as the duplication within the B clade also coincides with the loss of symbiotic interactions and mounting of glucosinolate defense responses, as well as the enhanced importance of pathogen elongation factor-derived elicitor recognition by EF-Tu (elongation factor thermo unstable) receptor (EFR) for defense response activation in Brassicaceae (Lacombe et al., 2010).

The *A. thaliana* EXO70B1 isoform is widely expressed throughout plant development. Despite this, the *exo70B1* loss-of-function (LOF) mutants lack prominent phenotypic deviations in younger stages (in contrast to the *exo70A1* mutant). The phenotype of *exo70B1* LOF mutation occurs later on adult rosette leaves (can occur earlier under the low light intensity of cultivation) in the form of hypersensitive response (HR) lesions caused by the salicylic acid (SA) hyperaccumulation. This phenotype, probably due to its later onset, is not accompanied by the overall growth retardation (under the high-light intensity, however, the rosettes may be smaller; Kulich et al., 2013; Pluhařová et al., 2019). The activation of the defense program in *exo70B1* mutants is dependent on the activity of the truncated nucleotide-binding and leucine-rich repeat (NLR) protein TIR-NBS2/TN2-*tn2* mutant, rescuing *exo70B1* mutants from HR (Zhao et al., 2015). The *A. thaliana* EXO70B2 isoform is expressed in seedling root tips and further in the development of the adult rosette upon biotic stress stimuli (Pečenková et al., 2020, 2011). The LOF mutants in the *EXO70B2* locus have no obvious developmental defects. Nevertheless, for mutants in both isoforms, a mild defect in stomatal functioning has been described—in the case of *exo70B1*, a retardation in light-induced opening, while in the case of *exo70B2*, a decreased stomatal closing reaction upon osmotic stress stimulation (Hong et al., 2016; Seo et al., 2016). While in seedlings and younger plants both isoforms support immunity, in later stages, probably with the onset of age- and SA-related resistance, the lack of EXO70B1 enhances the SA-dependent activation of HR lesions (Ortmannová et al., 2022; Pečenková et al., 2020, 2011; Stegmann et al., 2013, 2012; Wang et al., 2020; Zhao et al., 2015).

The relationship of the two isoforms has been studied using the corresponding double-mutant phenotype analysis and the cross-complementation approach. The two *LOF* mutations work in synergy on the level of root hairs, where they enhance prominently bacteria-stimulated root hair growth (Pečenková et al., 2020). Interestingly, EXO70B2 cannot complement the *exo70B1* HR phenotype (Marković et al., 2021). The significance of these findings and the functional relationship between the two isoforms remains largely unexplored.

It has been established that the exocyst complex plays a crucial role in the regulation of plant cell wall composition and architecture. The plant cell wall is a dynamic and adaptive structure, primarily composed of cellulose, hemicellulose, and pectin, particularly in *Arabidopsis* (Pauly and Keegstra, 2016). Several exocyst subunit mutants, including *sec8*, *sec15b*, and *exo70A1*, exhibit reduced pectin deposition in the seed epidermal cells, indicating the importance of exocyst-mediated vesicle trafficking for cell wall polysaccharide delivery (Kulich et al., 2010). Pectins are synthesized in the *cis*-Golgi and subsequently modified through methyl esterification (in medial- and *trans*-Golgi cisternae) and de-methyl esterification by pectin methylesterases (PMEs) in the apoplast (Atmodjo et al., 2011; Wolf et al., 2009; Zhang and Staehelin, 1992). In addition, pectins undergo acetylation via pectin acetyltransferases, which act antagonistically to pectin acetylesterases (PAEs) that remove acetyl groups (de Souza et al., 2014).

Beyond pectins, alterations in the levels or distribution of other cell wall components, including cellulose, lignin, and callose, have also been reported in various exocyst subunit mutants (Kalmbach et al., 2017; Kulich et al., 2018; Ortmannová et al., 2022; Vukašinović et al., 2017). Hemicellulose is another cell wall component that forms cross-links with cellulose microfibrils and includes xyloglucans, xylans, mannans, and glucomannans; these polysaccharides are also subject to *O*-acetylation by specific acetyltransferases, a process that influences their solubility and interaction with other wall polymers (Gille and Pauly, 2012; Scheller and Ulvskov, 2010; Schultink et al., 2015). Interestingly, XTH29, a member of the xyloglucan endotransglucosylase/hydrolase (XTH) family involved in remodeling hemicellulose, has been shown to colocalize with EXO70E2, a marker of unconventional protein secretion (UPS). This suggests a role for UPS pathways, potentially mediated by specific EXO70 isoforms, in hemicellulose deposition and remodeling (De Caroli et al., 2021; Wang et al., 2010).

It has also been observed that the exocyst complex functioning disruption provokes mislocalizations of mainly integral plasma membrane proteins (e.g., in *exo70A1*; Drdová et al., 2013; Kalmbach et al., 2017), but also vesicles crowding in cytoplasm (e.g., in *sec6* (Hématy et al., 2022) or *exo70B2* (Pečenková et al., 2011) or in paramural space in the case of *exo70B1* (Kulich et al., 2013).

Based on previous reports of defense-related defects in *Arabidopsis* LOF mutants of the EXO70B1 and EXO70B2 exocyst subunits, we investigated the underlying processes and causes of these phenotypes. First, we investigated whether the compromised defense responses observed in the EXO70B LOF mutants are also correlated with altered stomatal aperture states and changes in the cell wall composition. To better understand the overall regulatory physiological state of the mutants, we also performed RNA-seq analysis to identify the most significantly differentially expressed genes (DEGs) in the single *exo70B* mutants, as well as in the double mutant, at two developmental stages: seedlings and adult rosettes. Our analyses consistently revealed that the mutants, in comparison to wild-type (WT), have strongly altered stomatal dynamics and different modifications of cell wall pectin and hemicellulose. These phenotypes are likely directly mirrored in the defense-related phenotypic deviations and consequently contribute to the presence of primed defense gene expression patterns in the rosette leaves of mutant plants. These findings also provide insight into the degree to which the two evolutionarily relatively young EXO70B isoforms have functionally specialized/diversified.

## 2 Material and methods

### 2.1 Plant cultivation

For seedlings’ and plants’ cultivation, seeds were surface-sterilized (5 min in 70% ethanol, 2 × 5 min in 10% commercial bleach, rinsed three times in sterile distilled water) and stratified for 2–3 days at 4°C. Seeds were then germinated and grown on vertical 1/2 MS agar plates (half-strength Murashige and Skoog salts, Duchefa Biochemie, supplemented with 1% sucrose, vitamin mixture, and 1.6% plant agar, Duchefa Biochemie, Haarlem, Netherlands) at 21°C and 16 h of light per day for 5 to 7 days. For the cultivation of plants, 7-day-old seedlings were transferred into Jiffy Products International pellets and grown for 5 weeks at 22°C and 10/14 h of light per day in growth rooms. For each experiment, the following were used: *A. thaliana* T-DNA insertion mutants SAIL_339-D07 (*exo70B2-2* mutant) and GABI_114C03 (*exo70B1-1* mutant) obtained from NASC, Loughborough, United Kingdom and previously characterized in Kulich et al. (2013) and Pečenková et al. (2011), and the double mutant created via the crossing method. As a wild-type control, the Columbia-0 ecotype was used. Additionally, SALK_091877/*exo70B2-1* mutant and HR-complemented mutant *exo70B1*×*tn2* were used for supplementary experiments (Zhao et al., 2015).

### 2.2 Stomatal aperture analysis

Seven-day-old *in vitro* grown seedlings were flooded with stomata opening buffer (SOB) or SOB with *Pseudomonas syringae* non-virulent mutant in T3 secretion system *hrcC-* (*Pst hrcC-*) for 60 min. Mature leaves (4 weeks) were detached, submerged in SOB, and cultivated overnight to reach a steady state (Mock) condition prior to transfer to SOB with *Pst hrcC-* for 60 min as a treatment. To preserve the contribution of mesophyll response during the treatment, the epidermal cells were peeled immediately after the treatment/before observing. In addition, a treatment with fungal elicitor chitosan to mimic fungal pathogen attack was used to induce stomatal closure (stock 1%, 1,000× dilution). Both cotyledons and mature leaves were used, and the samples were treated for 30 min. To minimize bias, the experiments were performed in a double-blind setup: sample identities were concealed during imaging and quantification, apertures were measured, and only after statistical testing was sample identity revealed.

The length and width of the inner stomatal pore of every measurable stoma were measured; subsequently, young stomata with a length of less than 9 µm were filtered out. All experiments were repeated at least three times with the same trends and similar results (n > 150). For statistical analysis we used the Excel and ANOVA tests (p < 0.01) (https://astatsa.com/OneWay_Anova_with_TukeyHSD/).

Stomatal conductance was measured using a portable gas exchange system (LI-6400XT; LI-COR Biosciences, Lincoln, NE, USA). From 6-week-old plants grown under the conditions described above, fully expanded leaves (sixth or seventh from the apex) were collected. One leaf from each of six plants was clamped into the measurement chamber and allowed to equilibrate for 30 min to achieve steady-state gas exchange. The assay was initiated under ambient growth room conditions: CO_2_ concentration of 400 µmol mol^−1^, photosynthetically active radiation (PAR) of 120 µmol m^−2^ s^−1^, air temperature of 22°C, relative humidity of approximately 60%, and a flow rate of 500 µmol s^−1^. Subsequently, only the CO_2_ concentration was automatically reduced in two steps: first to 200 µmol mol^−1^ and then to 50 µmol mol^−1^. Between each CO_2_ step, a 30-min acclimation period was provided, followed by a 10-min data acquisition period, during which measurements were recorded every 30 s. Each measured leaf was photographed, and its surface area was determined using image analysis. Stomatal conductance values were then adjusted according to the measured leaf area. For statistical analysis and data presentation, the GraphPad (Prism) software was used.

### 2.3 Cell wall analysis

Treated (chitosan, 1,000× dilution of 1% stock, with Silwet, 0.005%) and mock (Silwet, 0.005%)-treated samples were submerged in 96% (v/v) ethanol and boiled at 70°C for 10 min. The pellets were collected by centrifugation (13,000 × *g* for 10 min) and dried in a speed vacuum concentrator at 30°C overnight. Samples were digested with 1 U/mg DW sample of *Aspergillus aculeatus* endo-polygalacturonase M2 (Megazyme, Bray, Ireland) in 50 mM ammonium acetate buffer (pH 5) at 37°C for 18 h to perform enzymatic fingerprinting of the cell wall. The oligosaccharides released from digestion were prepared and analyzed according to Paterlini et al. (2022). One milligram of alcohol-insoluble residue (AIR) was resuspended in 300 mL of 0.05 N NaOH and incubated overnight at 4°C. The suspension was then centrifuged at 13,000 × *g* for 15 min at 4°C. The resulting supernatant was used to quantify the released methanol after saponification, following the protocol described in Du et al. (2022). The pellet was washed twice with 70% ethanol and then dried. It was subsequently washed twice with 1% ammonium oxalate (pH 5.0). Then, 500 µL of 1% ammonium oxalate was added and incubated for 2 h at 80°C, followed by cooling to room temperature. After pectin extraction, uronic acid content was determined using the automated *m*-hydroxybiphenyl (MHBP) method (Thibault, 1979) and the method described by Ahmed and Labavitch (1978). A volume of 400 μL of freshly prepared 2 M trifluoroacetic acid (TFA) was added to the pellet. The samples were incubated at 120°C for 1 h, followed by centrifugation at 13,000 × *g* for 15 min at 4°C. The supernatant was removed, and the pellet was washed twice with 70% ethanol and then allowed to dry.

To determine the glucose content in the crystalline cellulose fraction, the TFA-insoluble pellet was hydrolyzed using 72% (v/v) sulfuric acid for 1 h at room temperature. After hydrolysis, the acid was diluted to a final concentration of 1 M with water, and the samples were incubated at 100°C for 3 h. Following incubation, the samples were filtered using 20-μm filter caps and analyzed using high-performance anion exchange chromatography with pulsed amperometric detection (HPAEC–PAD) on a Dionex ICS-5000 system (Thermo Fisher Scientific, Waltham, Massachusetts, USA), following the method described in Harholt et al. (2006).

Ruthenium red staining of seed coat pectins was performed according to McFarlane et al. (2014).

### 2.4 Microscopy and image analysis

De-esterified pectins were stained using COS-Alexa488 (Mravec et al., 2014). Seedlings that were 6-7-day-old were submerged in 1:2,000 diluted solution (1/2 liquid MS and COS-Alexa488) for 15 min, then washed for 2 min in 1/2 liquid MS, mounted onto slides, and observed using Zeiss 900 equipped with Airyscan 2 detector, C-Apochromat 63×/1.20 W Korr UV VIS IR FCS objective with 3% laser power (488 nm), and 4YxSR or 2YxSR multiplex Airyscan mode to achieve fast and precise scanning. Fine Z-stacks of individual stomata were captured (10 per cotyledon, at least two seedlings per genotype, two biological replicates). Airyscan processing was performed using the ZEN-Blue (Zeiss) software.

Image analysis was performed using the FIJI/ImageJ software (Schindelin et al., 2012). The mean intensity of COS-Alexa488 from the line profile cross-section of the stomatal average Z-stack projection was individually measured. For statistical analysis, the GraphPad (Prism) software was used.

The thickness of the seed coat stained with ruthenium red was quantified using FIJI/ImageJ. A segmentation model was trained using the Labkit plugin (Arzt et al., 2022) to identify the pectin-rich seed coat. The resulting binary images were manually curated to remove segmentation artifacts. The curated masks were then analyzed using the built-in Local Thickness plugin to calculate the mean thickness of the seed coat.

### 2.5 RNA isolation and RNA-seq analyses

RNA was isolated from young and adult (treated the same way as for cell wall analysis) *A. thaliana* plants using the RNeasy Plant kit (Qiagen, Hilden, Germany) according to the manufacturer’s instructions. Isolated RNAs were stabilized using GenTegra technology microtubes (GenTegra, Pleasanton, CA, USA). Strand-specific cDNA libraries were constructed from polyA-enriched RNA and sequenced on the Illumina NovaSeq 6000 platform with subsequent analysis performed by Eurofins. Rough reads were quality-filtered using Rcorrector and TrimGalore scripts with default parameters (Song and Florea, 2015; https://github.com/FelixKrueger/TrimGalore). The levels of transcript abundances [transcripts per million (TPM)] were determined using Salmon v.1.3.0 (Patro et al., 2017) with parameters –validateMappings, –seqBias, –gcBias, –posBias, –numBootstraps 30. Reference index was built from *A. thaliana*, TAIR10 cds library, v.20 101 214. Statistical evaluation and quality control of data analysis were performed using the sleuth R package, version 0.30.2 (Pimentel et al., 2017). Transcripts with q-value ≤ 0.05 and log_2_ fold-change ≥1 (upregulated) or ≤−1 (downregulated) were considered to be significantly differentially expressed. Gene Ontology analysis was conducted using DAVID bioinformatics resources (Huang et al., 2009). The visualization of the results of Gene Ontology (GO) analysis was conducted using the GOplot tool in R (Walter et al., 2015).

### 2.6 Protein extraction, Western blotting, and MAPK assay

Total protein extracts were isolated from 5–7-day-old *A. thaliana* seedlings according to the procedure described in Fernandez and Beeckman (2020). Briefly, seedlings of WT and *exo70B1*, *B2*, and double-mutant *exo70B1*×*B2* were ground in liquid nitrogen and dissolved in an extraction buffer containing PhosSTOP (Roche, Basel, Switzerland), and the concentration of proteins was determined using the Bio-Rad protein assay kit with bovine serum albumin (BSA) as the standard. The extracts were denatured by boiling in a 6× SDS loading buffer. The protein samples were separated using 10% Sodium dodecyl sulfate (SDS)–Polyacrylamide Gel Electrophoresis (PAGE) and analyzed via Western blotting using the α-p44/42-ERK antibody (SAB4301578), anti-MAPK3 (M8318, Sigma), anti-MAPK4 (A6979, Sigma), and anti-MAPK6 (A7104, Sigma Merck, Darmstad, Germany). The primary antibodies were incubated with the membranes for 3 h at room temperature in the blocking solution. Horseradish peroxidase-conjugated antibodies (anti-rabbit and anti-mouse, Promega Madison, Wisconsin, USA) were applied, followed by chemiluminescent Enhanced Chemiluminescent (ECL) detection (Amersham, Amersham, UK) using the Bio-Rad documentation system. Using the Gel Analysis function of ImageJ, signal intensities for protein bands were determined for each treatment from three different samples. Loading consistency was examined by staining the membrane with Ponceau S.

### 2.7 Text editing

OpenAI’s ChatGPT was used for language editing and refinement in the preparation of this manuscript.

## 3 Results

### 3.1 Defense-related phenotypes in *exo70B* mutants correlate with defective stomatal apertures

**Figure 1:**
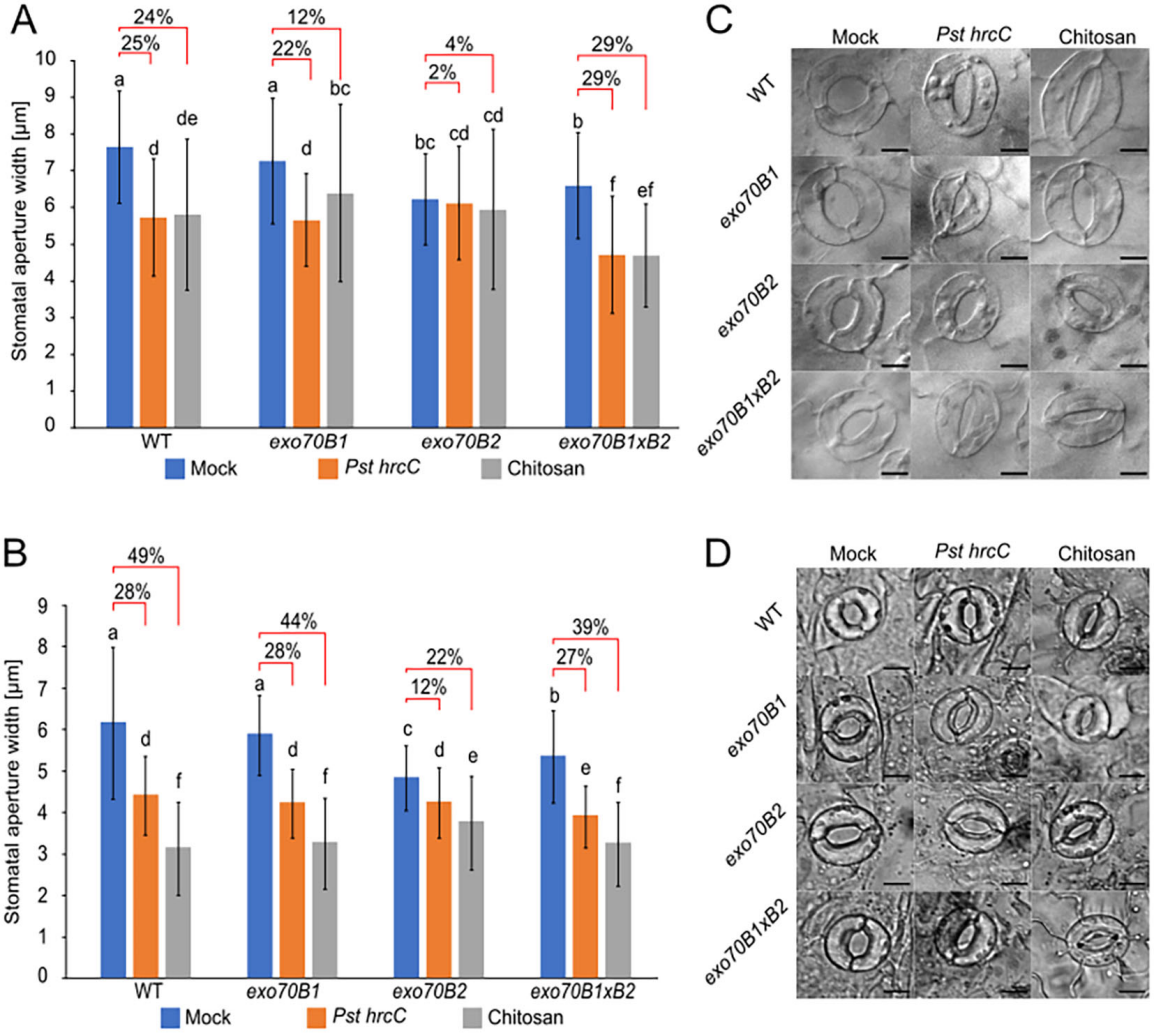
Stomatal aperture in *exo70B* mutants in comparison to WT. **(A)** Response of cotyledons (7-day-old plants) to bacterial *Pst hrcC-* or chitosan treatment. **(B)** Response of rosette leaves (4-week-old plants) to the same bacterial or chitosan treatment. **(C)** Stomata of cotyledon leaves treated with mock, *Pst hrcC-*, and chitosan. **(D)** Stomata of epidermal peels (4-week-old plants) treated with mock, *Pst hrcC-*, and chitosan. Single bars represent averages of widths of stomatal apertures ± SD. Red clamps represent relative decrease in stomatal aperture to mock treatment. One-way ANOVA with post-hoc Tukey’s Honestly Significant Difference (HSD) was used for statistics, p < 0.001, n > 150 per individual treatment; scale bar = 10 µm. Lowercase letters indicate statistical groupings.

To assess the role of stomata in the weakened defense capacity of young mutant plants *exo70B1*, *exo70B2* mutants, and double-mutant *exo70B1*×*B2*, compared to WT/Col-0 (Pečenková et al., 2020), we applied two types of treatments—one involving the inoculation of plants with non-virulent and mild defense-inducing bacterium *Pst hrcC-*, and the other one with chitosan elicitor derived from fungal cell walls. Since the *exo70B1* mutation in adult stages of development triggers the accumulation of SA, which modifies immune reactions, we tested both cotyledons and mature detached leaves prior to HR lesion development (Kulich et al., 2013); **Figure 1**).

First, we tested stomatal response in cotyledons soon after germination. In mock controls, there were clear differences in the initial stomatal aperture–stomata of *exo70B2* mutant (and to a lesser extent also of *exo70B1*×*70B2* double mutant), which were significantly almost 20% more closed compared to WT and *exo70B1* lines after the mock treatment (**Figures 1A, C**). Both *Pst hrcC-* and chitosan treatments induced stomatal closure in all studied lines, but the single *exo70B2* mutant exhibited the weakest stomatal closure response compared to its mock condition. When the stomatal aperture under the mock treatment was set as 100%, it was found that the WT stomata closed for 25% for *Pst hrcC-* and 24% for chitosan, *exo70B1* for 22% and 12%, and *exo70B2* for only 2% and 4%. The most interesting double-mutant *exo70B1*×*B2* stomata were the most closed post-treatment. However, compared to its initial state, the response range was “rescued” to the levels found for WT, with 29% for both treatments (**Figure 1C**).

In the case of rosette leaves, WT and single *exo70B1* mutant share similar behavior (28%, 49% WT, 28%, and 44% *exo70B1*), *exo70B2* responds more strongly when compared to the cotyledon experiment (12% and 22%), and again, the rescue phenotype of the double mutant was present, making it more similar to WT than single mutants (**Figures 1B, D**). In addition, there were clear differences in response to mock treatments—stomata of *exo70B2* mutant were significantly more closed compared to all other lines after the mock treatment.

**Figure 2:**
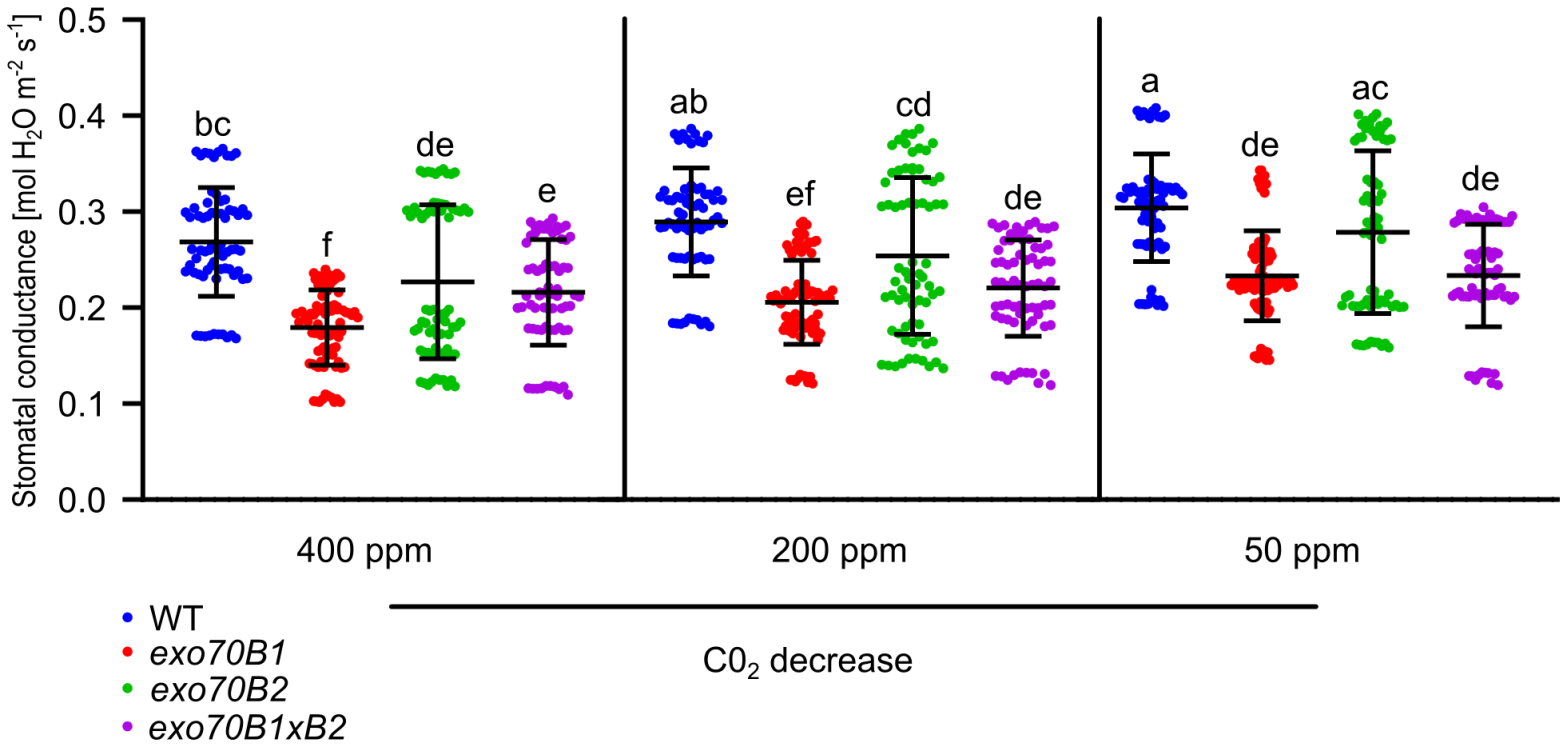
Stomatal conductance in *exo70B* mutants in comparison to WT. Increasing stomatal conductance in WT and mutants after ambient CO_2_ decrease. Conductances of leaves from 6-week-old plants were individually measured at CO_2_ steady-state ambient conditions of 400, 200, and 50 ppm. One-way ANOVA with post-hoc Tukey’s HSD was used for statistics, p < 0.05, n > 5 independent leaves per genotype; error bars represent SD. Conductance is defined as mol H_2_O m^−2^ s^−1^. WT, wild type. Lowercase letters indicate statistical groupings.

Based on the results pointing to different initial stomatal aperture states under the mock condition, the conductance of biotically non-challenged plants was additionally measured using the gas analyzer LI-COR 6400XT. To complement defense-related stomatal closure data and to promote stomatal opening, a sequential decrease in CO_2_ concentration was employed. The conductances were measured and presented as absolute values in graphs (**Figure 2**). In this type of assay, stomata of mutant lines were significantly less conductive under normal conditions, as well as under the conditions of CO_2_ decrease. These trends were also confirmed when an HR-complemented double-mutant *exo70B2*×*tn2* and another *exo70B2* line (*exo70B2-1*) were used (**Supplementary Figure 1A**).

Overall, our results confirm that a portion of defense-related phenotypical deviations in *exo70B* mutants, and especially in the case of younger *exo70B2* plants, was caused by defects in their stomatal aperture states.

### 3.2 Cell wall of *exo70B* mutants is differentially modified

Altered defense responses in *exo70B* mutants were also observed in experiments that did not involve pathogen entry via stomata (e.g., Ortmannová et al., 2022; Pečenková et al., 2011). To assess whether the observed phenotypic changes are then related to the modifications in cell wall composition, we performed an analysis of *exo70B* mutants’ cell walls using enzymatic fingerprinting. For all mutant lines, mock-treated and chitosan-treated adult rosette leaves were analyzed and compared with WT plants (Drs et al., 2025).

**Figure 3:**
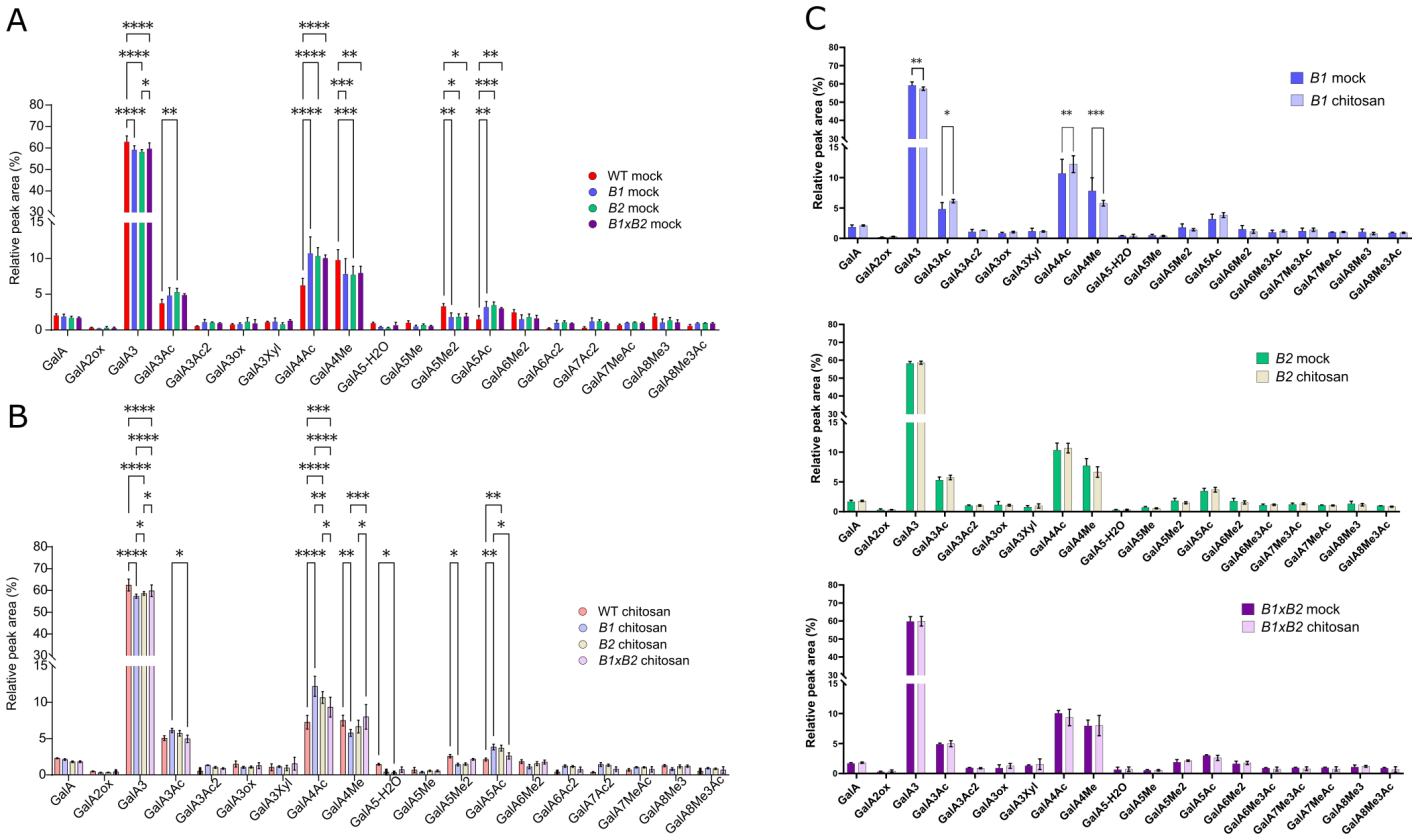
The *exo70B* mutants had altered pectin modifications by methylation and acetylation under both mock and chitosan treatment conditions. **(A)** Enzymatic fingerprinting of homogalacturonans; the mutant lines have more of de‐methyl-esterified and acetyl-esterified pectins, as evidenced by the decrease in relative amounts of GalA4Me and increase in GalA3Ac, respectively. **(B)** Similar trend was also found for chitosan-treated plants. **(C)** In comparison of mock- and chitosan-treated plants for each line, significant differences were found only for *exo70B1*. OGs are named GalAxMeyAcz. x, y, and z indicate the degree of polymerization (DP) and the number of methyl and acetyl ester groups, respectively. GalA, galacturonic acid; Me, methyl ester group; Ac, acetyl ester group. Data represent mean ± SD, n = 4, *p < 0.05, **p < 0.01, ***p<0.001; ****p<0.0001, Student’s t‐test.

No significant differences in cell wall glucose composition were detected (**Supplementary Figure 1B**). However, the levels of digestible pectin modifications—specifically methylation and acetylation—were found to be significantly altered in mutants (**Figure 3**). All mutants displayed a reduction in methyl-esterified digestible pectins and an increase in acetyl-esterified digestible pectins compared to WT (**Figure 3A**). Furthermore, in chitosan-treated WT plants, there was a significant increase in de-methylated and acetyl-esterified pectins compared to mock (**Figure 3B**; Drs et al., 2025). This trend was also observed in the *exo70B1* mutant, while no significant changes were detected in *exo70B2* and *exo70B1*×*B2* double mutants between their respective mock and chitosan treatments (**Figure 3C**). Similar to the mock treatment, chitosan-treated *exo70B* mutants exhibited less methyl-esterified and more acetyl-esterified pectins.

Additionally, hemicellulose composition slightly differed in the mutants compared to WT, following both mock and chitosan treatments, most prominently in the case of the *exo70B2* mutant. This was reflected in the enhanced acetylation of some hemicellulose motifs and the decreased acetylation of others; similar to the situation with pectins, except for *exo70B1*, no significant changes in chitosan versus mock-treated plants were uncovered Fry et al., 1993 (**Supplementary Figures 2A, B**).

**Figure 4:**
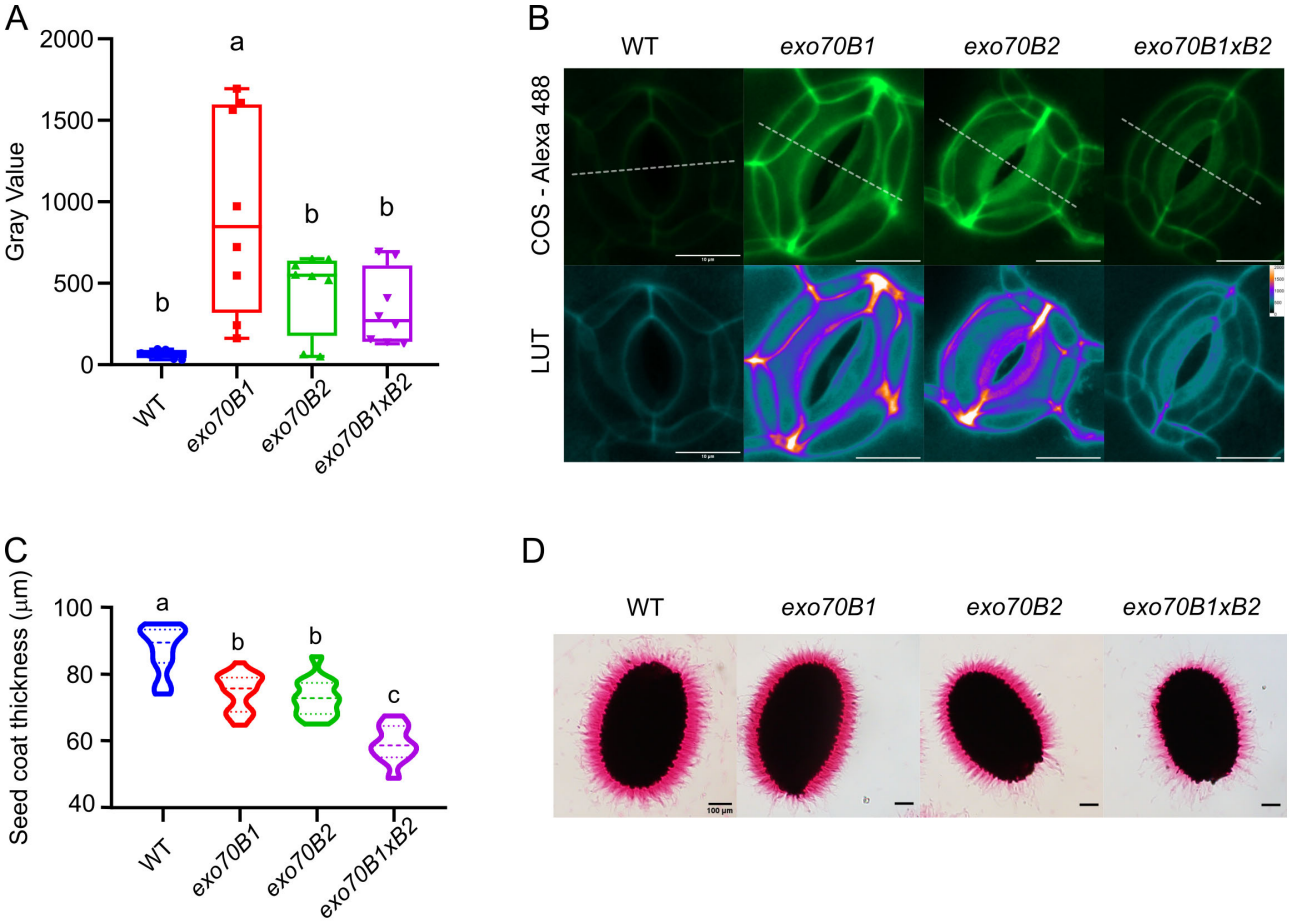
Pectin modifications are observable on microscopic level. **(A)** Analysis of de-esterified pectins of stomata (in cotyledons) stained with COS-Alexa488. Graph represents the mean gray value of the line cross-section of the average Z-stack projection. The one-way ANOVA with post-hoc Tukey’s HSD test (p < 0.001, n = 8 stomata per genotype). **(B)** Average projections of COS-Alexa488-stained stomata; magenta dashed line represents the cross-section for the measurement; the second “LUT” row shows more precisely the difference of the signal and distribution of de-esterified pectins between the WT and mutants using specific look-up-table (LUT) for the visualization (scale bar = 10 µm). **(C)** Seed coat pectin analysis. Plot shows the average thickness of ruthenium red-stained pectin coating. Statistical analysis was performed using one-way ANOVA with post-hoc Tukey’s HSD test (p < 0.001, n > 30 seeds per genotype). **(D)** Ruthenium red-stained seed coat pectins of the studied lines (WT, *exo70B1*, *exo70B2*, and *exo70B×B2*; scale bar = 100 µm). WT, wild type. Lowercase letters indicate statistical groupings.

Based on these results, we wanted to inspect whether the overall cell wall modification defects are also responsible for the altered stomatal function. We examined the pectin methylation status of stomatal cell walls using the COS probe, which predominantly binds to de-esterified pectins (Mravec et al., 2014). We found that the COS-Alexa488 labeling intensity in *exo70B* mutant was higher, indicating a larger proportion of de-esterified pectins in the stomatal cell walls of the mutants (**Figure 4A**). To verify if the altered pectin modification may be relevant for other developmental stages, we performed ruthenium red staining of seed coat pectins. Consistent with the pectin analysis in adult tissues and other exocyst mutants, we observed differences in the thickness of the hydrated extruded seed coat pectin mucilage in both *exo70B1* and *exo70B2* mutants, with an even more pronounced effect in the *exo70B1×B2* double mutant (**Figure 4B**).

To conclude, *exo70B* mutants exhibited altered modifications of cell wall pectins and minor changes in hemicellulose. The most prominent deviations in pectin modifications were observed for *exo70B1*, while the *exo70B2* mutant showed more changes in hemicellulose composition.

### 3.3 RNA-seq analyses of *exo70B* mutants reveal differential defense- and cell wall-related gene expression

In order to investigate the more general genetic context of *Arabidopsis* EXO70B isoform and *exo70B* observed phenotypes, we performed RNA-seq analysis of *LOF* mutants in these genes. We acquired the RNA for evaluation via two approaches, from 7-day-old seedlings vertically grown *in vitro* and from 5–6-week-old adult plants grown in jiffy pellets, before the first HR phenotypes started to appear, always for the two *exo70B* mutant lines, the double mutant, and the WT control, either mock- or chitosan-treated (**Supplementary Tables 1**-**3**).

The seedlings’ RNA-seq analysis replicates had high correlation coefficients, as demonstrated by the heatmap (**Supplementary Figure 3A**). When the criteria for the identification of significant changes in gene expression (DEGs) were set as q value <0.05 and log_2_ (fold-change) >1, a total of 688, 288, and 181 DEGs were identified for seedlings of *exo70B1*, *exo70B2*, and the double mutant, respectively, in comparison to the wild-type control. There were prominent overlaps among the mutants’ sets of DEGs, with *exo70B1* having a higher proportion of specific DEGs (**Supplementary Figure 3B**). A list of significantly up- and downregulated genes for each line is provided in **Supplementary Table 2**.

The RNA-seq for adult rosette leaves revealed significant 885 DEGs for *exo70B1*, 1,759 for *exo70B2*, and 1,547 for the double *exo70B1*×*B2* mutant (**Supplementary Figure 4A**). Interestingly, in contrast to the seedling stage, the adult *exo70B* LOF mutants had a highly similar overall transcription pattern and fewer unique DEGs. The absence of clear differences in overall gene expression patterns suggests, despite potential differential tissue expression, the incompleteness of the specialization process between the two isoforms, in agreement with the evolutionary novelty of this isoform’s clade bifurcation. A list of significantly up- and downregulated genes for each line in the adult stage is provided in **Supplementary Table 3**.

To uncover differential gene expression under biotic stress-induced conditions, RNA-seq analysis was also performed for chitosan-treated adult plants, thus revealing the differential intensity of the activation of chitosan-related responses in mutant lines, compared to chitosan-treated WT. There were 1,778, 2,105, and 1,910 DEGs found for *exo70B1*, *exo70B2*, and the double mutant, respectively, compared to chitosan-treated wild-type control (**Supplementary Figure 4B**, **Supplementary Table 2**). The heatmap of sequencing replicates confirms consistent expression patterns for all studied lines and treatments (**Supplementary Figure 4C**).

To understand in-depth the processes affected by LOF of *exo70B1*, *B2*, and *B1*×*B2*, we performed GO analysis for sets of DEGs. The representative gene categories for each line, upregulated and downregulated, seedlings and adults, are shown in **Supplementary Tables 2** and **3**. We then focused our attention on the selection of GO categories that were found to be significantly and prominently enriched and statistically well supported for most of the studied mutant lines and treatments such as responses to bacteria, cell wall organization, water stress, and salicylic and jasmonic acids in case of seedlings, as well as overall defense capacity and signal transduction in case of adult plants (**Table 1**). In agreement with EXO70Bs’ predicted regulatory function in secretory pathways, the enrichment in DEGs associated with plasma membrane and apoplast GO categories was found as well, and also, surprisingly, an enrichment in genes assigned to the chloroplast compartment GO category (**Supplementary Figures 5**-**10**). The same categories of genes were enriched more prominently in *exo70B* mutants than in the WT when the chitosan- and mock-treated expression patterns for each line were compared (**Supplementary Table 3**, **Supplementary Figures 11**-**14**).

**Table 1:** Selected GO categories used for the analysis of *exo70B* DEGs.

Seedlings		
GOTERM_BP_DIRECT	GO:0009753	Response to jasmonic acid
GOTERM_BP_DIRECT	GO:0009617	Response to bacterium
GOTERM_BP_DIRECT	GO:0009751	Response to salicylic acid
GOTERM_BP_DIRECT	GO:0071555	Cell wall organization
GOTERM_BP_DIRECT	GO:0009414	Response to water deprivation
GOTERM_BP_DIRECT	GO:0009737	Response to abscisic acid
Adult plants		
GOTERM_BP_DIRECT	GO:0006952	Defense response
GOTERM_BP_DIRECT	GO:0007165	Signal transduction
GOTERM_BP_DIRECT	GO:0009738	Abscisic acid-activated signaling pathway
GOTERM_MF_DIRECT	GO:0004676	3-Phosphoinositide-dependent protein kinase activity
GOTERM_CC_DIRECT	GO:0005886	Plasma membrane
GOTERM_CC_DIRECT	GO:0009505	Plant-type cell wall
GOTERM_CC_DIRECT	GO:0048046	Apoplast
GOTERM_CC_DIRECT	GO:0009507	Chloroplast

GO, Gene Ontology; DEGs, differentially expressed genes.

Our RNA-seq analysis, as anticipated, further confirms the involvement of EXO70Bs in cell wall- and defense-related processes.

### 3.4 Stomata- and cell wall-related DEGs found for *exo70B* mutants

**Figure 5:**
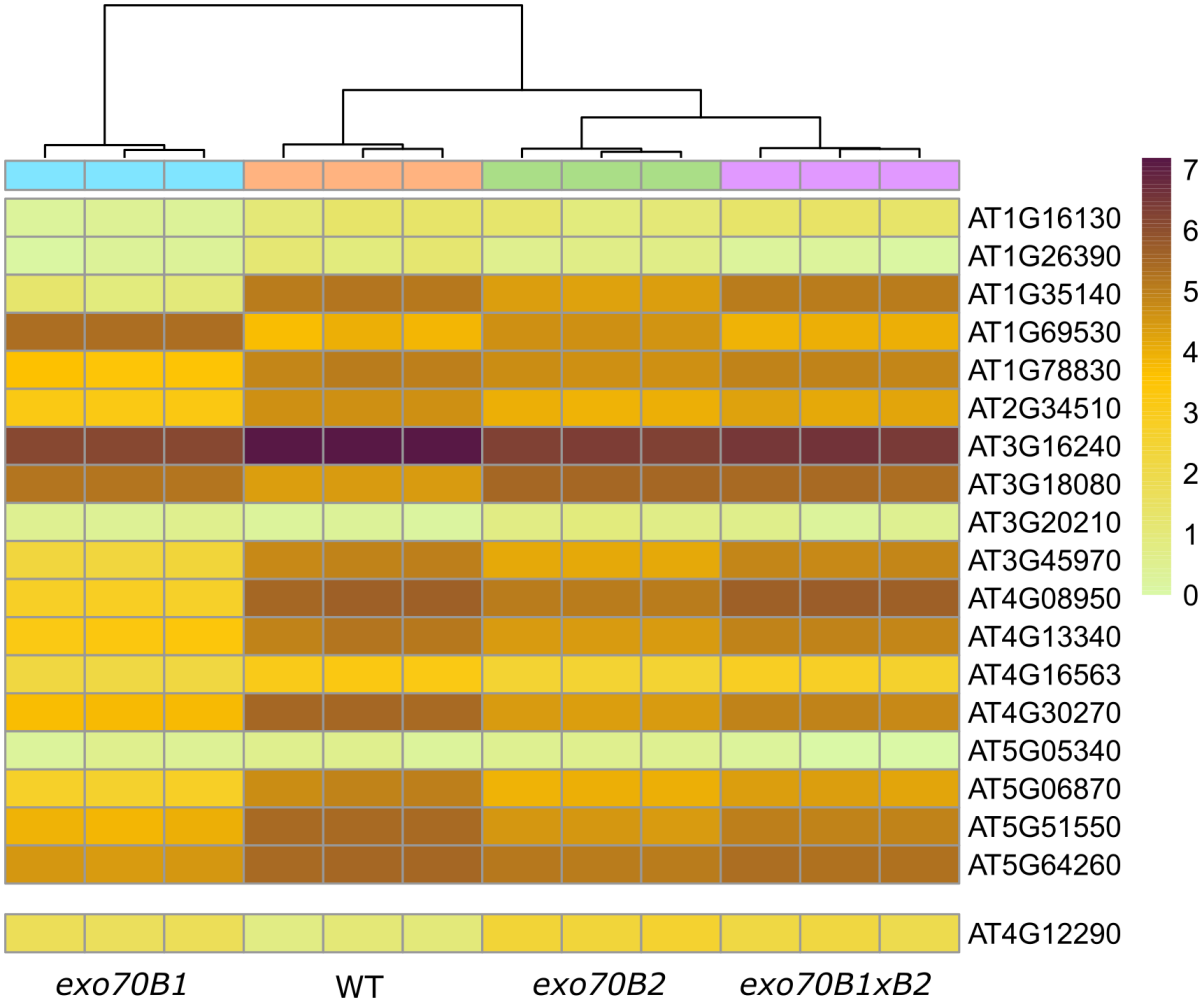
A heatmap displaying transcription levels (lnTPM) of significantly differentially expressed genes from GO categories plant cell wall (GO:0009505, upper heatmap) and stomatal movement (GO:0010118, lower heatmap with one gene found) in seedlings of all mutant genotypes vs. WT (q ≤ 0.05, fold-change ≥2). GO, Gene Ontology; WT, wild type.

**Figure 6:**
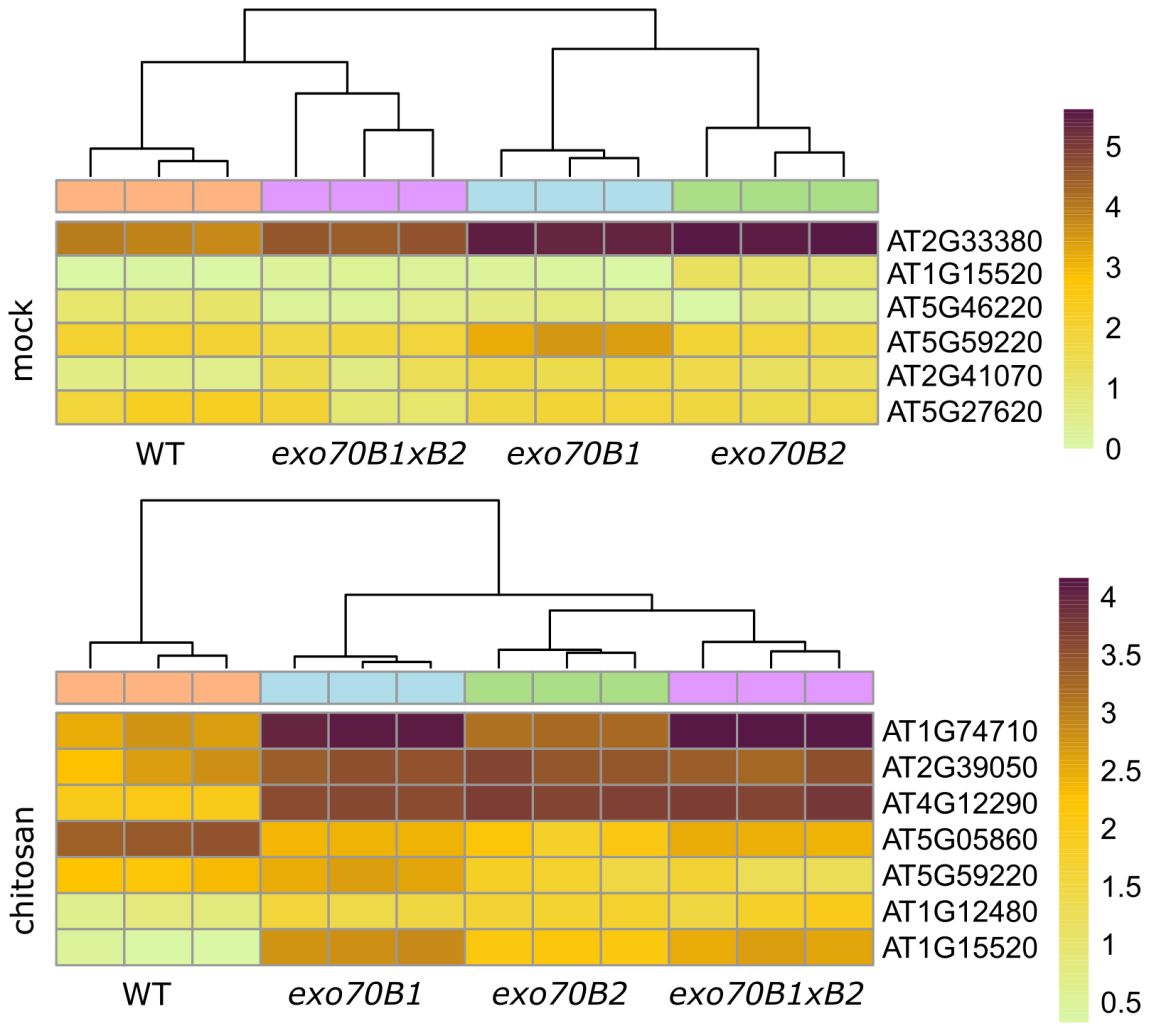
A heatmap displaying transcription levels (lnTPM) of significantly differentially expressed genes from GO category stomatal movement (GO:0010118) in all mutant genotypes vs. WT, for adult plants, mock (upper heatmap), and chitosan (lower heatmap) treatments (q ≤ 0.05, fold-change ≥2). GO, Gene Ontology; WT, wild type.

**Figure 7:**
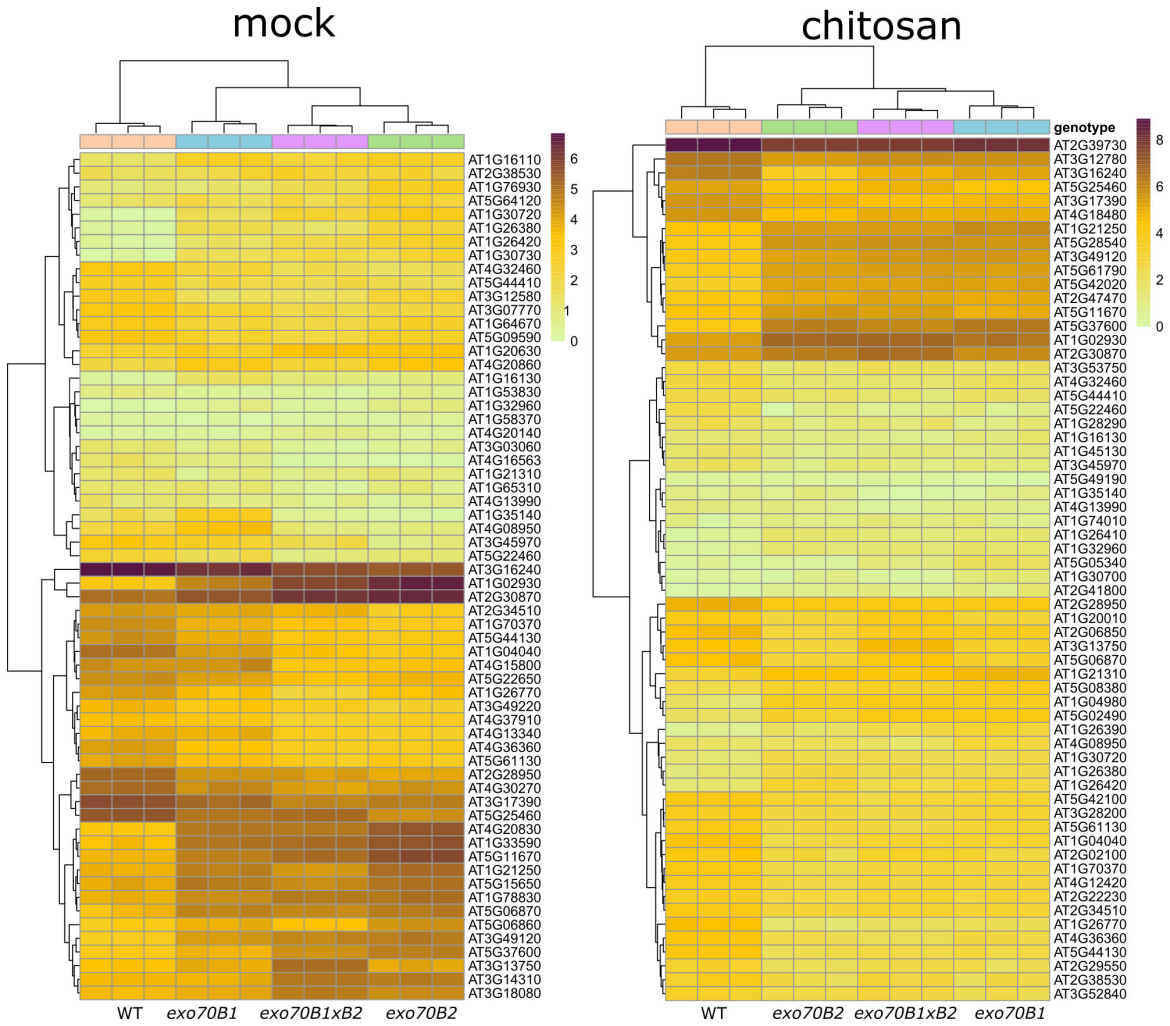
A heatmap displaying transcription levels (lnTPM) of significantly differentially expressed genes from GO category cell wall (GO:0009505) in all mutant genotypes vs. WT, for adult plants, mock (on the left), and chitosan (on the right) treatments (q ≤ 0.05, fold-change ≥2). GO, Gene Ontology; WT, wild type.

We also looked specifically into the activity of genes that may be involved in reported phenotypes and assigned to the two gene categories—plant cell wall (GO:0009505) and stomatal movement (GO:0010118). Many interesting genes involved in plant cell wall functions were identified to be differentially expressed in *exo70B*s in the seedling stage, but only one candidate gene, a vacuolar amine oxidase that may be involved in stomatal function via Reactive Oxygen Species (ROS) production (At4g12290; **Figure 5**). Furthermore, for adult plants, several stomata-relevant genes were found to have altered expression in *exo70B*s, either upon mock or chitosan treatment (**Figure 6**), and many more cell wall function-related candidates were identified (**Figure 7**). The role of the most prominently deregulated genes in reported stomatal and cell wall phenotypes remains to be inspected individually in the future.

In order to understand the alterations in pectin modifications observed in the cell wall analysis of *exo70B* mutants, we specifically and separately searched for candidate genes potentially responsible for these changes. Anticipating defective secretion as the cause of these phenotypes, starting from the earliest stages of development, we focused on the seedling dataset and identified several genes involved in pectin modifications that were differentially regulated in *exo70B* mutants, in comparison to WT (**Table 2**; log_2_FC values greater or smaller than 1 are indicated in bold). At the same time, **Table 2** also highlights candidate genes that may contribute to the compensatory effects of the two *exo70B* mutations, as their expression levels in the double-mutant *exo70B1*×*B2* were the most similar to those observed in the wild type (e.g., At1g60590, At2g43870, and At4g19420). However, it remains to be determined whether these genes represent primary factors underlying the mutants’ phenotypes.

**Table 2:** Pectin modification-related genes.

Gene ID	Description	B1s	B2s	B1×B2s
AT1G05650	Pectin lyase-like superfamily protein	**1.08**	0.52	0.27
AT1G48100	Pectin lyase-like superfamily protein	**1.22**	0.52	−0.23
AT1G60590	Pectin lyase-like superfamily protein	**1.38**	**1.40**	0.55
AT2G43870	Pectin lyase-like superfamily protein	**1.05**	0.31	0.04
AT3G10720	Plant invertase/pectin methylesterase inhibitor superfamily protein	**−1.14**	−0.19	−0.06
AT4G19420	Pectin acetylesterase family protein	**−1.13**	−0.71	−0.47
AT5G62330	Plant invertase/pectin methylesterase inhibitor superfamily protein	−0.44	**−1.93**	−0.37
AT5G62360	Plant invertase/pectin methylesterase inhibitor superfamily protein	0.62	**1.25**	**1.31**

Genes with significant differences in transcription levels (log_2_FC higher or smaller than 1 (in bold), q-val ≤ 0.05, for at least one of the lines) for several pectin-modifying enzymes in seedlings; six genes were found for *exo70B1*, three for *exo70B2*, and only one for the double-mutant *exo70B1*×*B2*. The milder and mostly insignificant changes found for the double mutant are indicative of the involvement of this set of enzymes in compensational effect of the two *exo70B* mutations.

s, seedlings.

Based on the results of our guard cell aperture and cell wall analyses, as well as the results of pectin staining of stomatal cell walls, we next examined whether we can, using our RNA-seq data, identify genes responsible for cell wall modification defects observed in *exo70B*s’ stomata. To address this, we compared the list of genes identified via single-cell RNA-seq analysis and found an overlap in genes with significantly altered expression levels (log_2_FC > |1|, in bold; q ≤ 0.05 for at least one of the lines) within the “cell wall” category (GO:0009505) and the stomatal gene set determined in the single-cell RNA-seq experiment by Peng et al. (2024). Only four genes were detected (**Table 3**), including At1g30720 and At1g64670, which encode enzymes involved in cuticle and lignin formation, respectively (Daniel et al., 2015; Jakobson et al., 2016). Notably, At1g64670 was more strongly downregulated in the double mutant than in the single mutants. Interestingly, all four genes were found to be less deregulated and closer to wild-type levels following chitosan treatment. The relatedness of these genes with pectin-modification states remains to be further inspected.

**Table 3:** The list of genes identified through the overlap of genes found in a stomatal lineage single-cell RNA-seq analysis (Peng et al., 2024) and cell wall-related genes (GO: 0009505) with significantly altered expression levels found in our RNA-seq analysis.

Gene ID	Description	B1a	B1ach	B2a	B2ach	B1×B2a	B1×B2ach
AT1G30720	FAD-binding Berberine fam. prot.	**3.36**	**1.20**	**4.75**	**1.17**	**4.24**	**1.06**
AT1G33590	Leucine-rich repeat (LRR) fam. prot.	**1.56**	*ns*	**2.12**	*ns*	**1.68**	0,13
AT4G20830	FAD-binding Berberine fam. prot.	**1.83**	0.88	**2.41**	0.69	**1.75**	0.53
AT1G64670	Alpha/beta-hydrolases superfam. prot.	−0.66	−0.83	−0.42	−0.60	**−1.02**	−0.79

log_2_FC higher or smaller than 1 (in bold); q-val ≤ 0.05 for at least one of the lines. The two genes, At1g30720 and At1g64670, are involved in leaf epidermal cuticle formation (Daniel et al., 2015; Jakobson et al., 2016); the latter is more prominently downregulated in the double mutant than in single ones. Interestingly, with exception of downregulated At1g64670, these genes were found to be expressed on more wild-type (WT)-like levels upon chitosan treatments.

a, adult; ch, chitosan; ns, not significant.

Although we did not identify canonical stomatal movement components (such as channels and pumps) among the most prominently altered genes, we examined their expression levels and identified several that showed significant changes in at least one of the analyzed *exo70B* mutants (**Table 4**). We found that these changes were generally mild but became more pronounced and significant after chitosan elicitor treatment. This suggests that the observed effects likely represent secondary alterations as a consequence of defense-related responses, rather than primary changes in proton pump expression levels. We also addressed the possibility that the alterations in stomatal functions may be the consequence of altered ABA signaling, in accordance with our GO analysis results. We looked specifically into the overlap of genes involved in response to abscisic acid (GO:0009738) and those found to be significantly differentially expressed in *exo70B* mutants (**Supplementary Table 4**). Interestingly, the highest number of DEGs and the highest ranges of changes were detected in the case of *exo70B1*, and not in the case of *exo70B2*, which has the most deviated stomatal phenotype in the seedling stage.

**Table 4:** The list of genes encoding for canonical stomatal movement components found to have significant changes in expression levels in at least one of the analyzed *exo70B* mutants.

Gene ID	Symbols	Description	B1s	B1a	B1ach	B2s	B2a	B2ach	B1×B2s	B1×B2a	B1×B2ach
AT1G12480	SLAC1	C4-dicar./malic tran.	0.43	*ns*	**1.05**	*0.23*	0.87	**1.33**	*ns*	0.77	**1.37**
AT4G17970	ALMT12	Al-activ., malate t. 12	−0.17	0.51	0.25	*ns*	−0.6	−0.25	−0.27	−0.56	−0.81
AT2G18960	AHA1	H(+)-ATPase 1	−0.12	−0.44	−0.12	*ns*	−0.21	−0.14	*ns*	−0.21	−0.19
AT4G30190	AHA2	H(+)-ATPase 2	0.13	−0.44	*ns*	*ns*	−0.21	0.24	*ns*	−0.35	*ns*
AT5G57350	AHA3	H(+)-ATPase 3	−0.15	0.24	0.1	−0.08	0.49	0.19	−0.13	0.43	0.24
AT3G47950	AHA4	H(+)-ATPase 4	*ns*	*ns*	0.87	−0.29	*ns*	0.97	*ns*	*ns*	0.83
AT1G17260	AHA10	H(+)-ATPase 10	nd	*ns*	*ns*	nd	*ns*	**1.1**	nd	*ns*	*ns*
AT5G62670	AHA11*	H(+)-ATPase 11	−0.27	−0.69	**−1.08**	−0.35	−0.89	**−1.55**	−0.21	−0.85	**−1.31**
AT5G46240	KAT1	Potassium channel 1	−0.69	*ns*	0.55	−0.47	*ns*	−0.32	−0.33	*ns*	*ns*
AT4G18290	KAT2	Potassium channel 2	0.39	*ns*	*ns*	*ns*	*ns*	0.49	*ns*	*ns*	0.7

Difference of transcription levels (log_2_FC) for several key regulators of stomatal movement, where significant difference (q-val ≤ 0.05) for at least one comparison (mutant vs. wild-type) was found. More prominent changes (log_2_FC higher or smaller than 1) are marked in bold. For some genes, trends in expression levels are more severe in adult stage (a), or in adult after chitosan treatment (ach), compared to seedlings (s; e.g., AHA11). In others, rather compensatory gene regulation occurs after chitosan (e.g., AHA1 and AHA3). Several stomata-specific genes in *exo70B* mutants are activated only by the chitosan treatment (e.g., AHA4 and KAT1 in *exo70B1*, AHA4 and AHA10 in *exo70B2*, and KAT2 in *exo70B1×B2*). The AHA10 is thus a possible candidate target for a phenotype correction in the double mutant in comparison to *exo70B2*.

s, seedlings; a, adult; ch, chitosan; nd, not detected (expression level too low); ns, not significant; * expression in guard cells not confirmed.

Thus, the stomata-focused analysis of our RNA-seq data confirms the importance of EXO70Bs for stomatal cell wall, in addition to pectins, also for other components (e.g., cuticle) and possibly through cross-talk with defense participants that may affect the cycles of stomatal opening and closing.

### 3.5 Differences in DEGs among *exo70B* mutants are more pronounced at the seedling stage, while in adult plants, they converge toward spontaneous transcriptional defense activation

**Figure 8:**
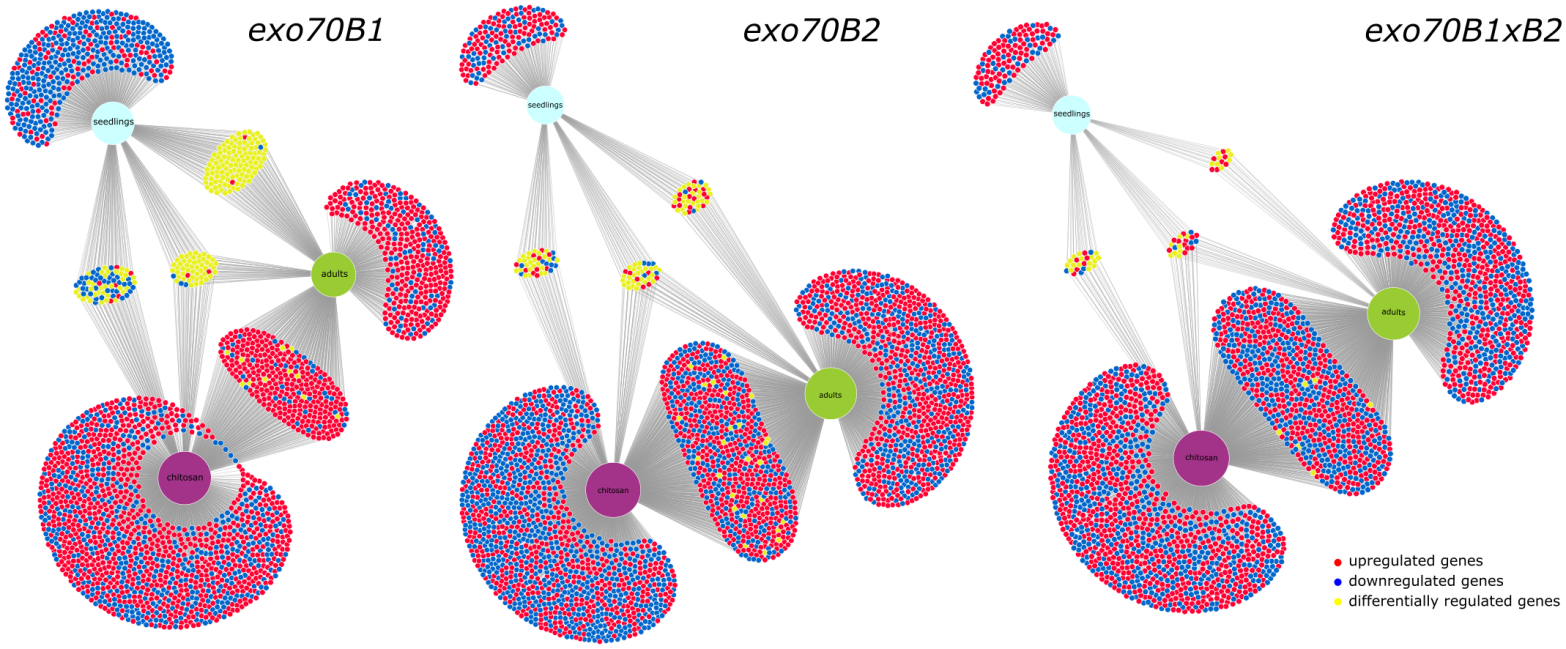
DiVenn diagrams demonstrate overlaps and differences in sets of differentially expressed genes (DEGs) determined for seedling stages and adult developmental stages, mock- and chitosan-treated for each of the analyzed *exo70B* lines; more unique DEGs for seedling stage are observable for *exo70B1* compared to *B2* and double-mutant *B1×B2*, indicating later convergent transcriptional reprogramming.

To investigate the transcriptional basis of the reported stage-dependent differences in immune responses of *exo70B1* and *exo70B2* mutants, we compared global gene expression profiles between seedling and adult developmental stages, mock, and chitosan treatment. The comparative analysis revealed more DEGs unique to the seedling stage in the case of *exo70B1* compared to *B2* and double-mutant *B1*×*B2* (**Figure 8**). In particular, the sets of DEGs showed limited overlap between the two stages, indicating that substantial transcriptional reprogramming may occur during plant development. This also suggests a compensatory effect emerging later in development that may normalize or attenuate early gene expression imbalances.

Unlike in seedlings, in adult plants, both *exo70B1* and *exo70B2* mutants exhibited a strong upregulation of genes involved in defense responses, suggesting a state of constitutive transcriptional activation reminiscent of stress-induced conditions (**Supplementary Tables 2**, **3**). This observation was further supported by the similarity in expression profiles between the mutants and WT plants treated with chitosan (see **Supplementary Figure 4**). These findings imply that *exo70B* mutants exist in a primed or alert transcriptional state, potentially mimicking responses triggered by exogenous stimuli. To explore the possible mechanistic basis of this transcriptional activation, we examined the mitogen-activated protein kinase (MAPK) cascade, a key component of plant defense signaling (Westman et al., 2019). However, no significant differences were observed in either the basal activation or elicitor-induced phosphorylation of MAPKs in the mutants compared to WT (**Supplementary Figure 15**). Interestingly, in *exo70B2*, the MAPK3 gene showed elevated transcript levels, possibly reflecting disrupted negative feedback or altered signal integration due to the loss of functional EXO70B2–MAPK3/6 interactions (Brillada et al., 2021). Nevertheless, this increase in transcript abundance did not translate into altered phosphorylation patterns or activation dynamics.

Overall, our data suggest that the developmental stage plays a key role in shaping the transcriptional landscape of *exo70B* mutants. In early stages, the mutations resulting in cell wall and stomatal deviations, in combination with initial consequential gene expression in later stages, lead to the likely compensatory activation of defense responses.

## 4 Discussion

In this study, we demonstrate that the two *A. thaliana* EXO70B isoforms B1 and B2, previously characterized primarily for their roles in plant immunity, also contribute to stomatal dynamics. Specifically, we show that *exo70B1* and *exo70B2* mutants exhibit impaired stomatal closure following pathogenic bacteria or fungal elicitor exposure, as well as a compromised state of openness under normal conditions, or opening response under the conditions of reduced atmospheric CO_2_. In contrast to previous studies (Hong et al., 2016; Seo et al., 2016) that reported no effects of mock treatment on *exo70B2*, our data reveal that *exo70B2* mutants exhibited partially closed stomata even after mock buffer treatment, suggesting an increased sensitivity to mechanical or osmotic stimuli (**Figure 1**). This phenotype was observed in both intact cotyledons and epidermal peels from 4-week-old rosette leaves, indicating that EXO70B2 functions in a broader context beyond immune signaling and participates in the regulation of stomatal behavior under routine physiological stimuli.

Beyond their role in stomatal control, EXO70B1 and EXO70B2 also contribute to cell wall remodeling under both basal and stress conditions. While EXO70A1 primarily affects cellulose deposition (Vukašinović et al., 2017), *exo70B* mutants do not have significantly altered cellulose content, as reflected by unchanged glucose levels. Instead, our analyses point to a role in modifying pectin and hemicellulose. These cell wall polymers are central to defense, with pectin methylesterases, acetylesterases, and their inhibitors acting as critical regulators of pathogen resistance (Bethke et al., 2014; Del Corpo et al., 2020; Lionetti et al., 2017, 2007; Pogorelko et al., 2013; Vogel et al., 2004; Voxeur et al., 2019).

Previous work has identified the exocyst complex as essential for pectin deposition in the seed coat (Kulich et al., 2010). Our results extend these findings to leaf tissue, specifically showing that EXO70B1 and EXO70B2 also affect pectin modifications there. We propose that EXO70Bs regulate the modification of pectins through the canonical exocyst complex, which includes core subunits such as SEC8, known to mediate vesicle tethering and pectin deposition (Kulich et al., 2010). Established protein–protein interactions reported between EXO70Bs and other exocyst subunits (Kulich et al., 2013; Pečenková et al., 2011) support this view. Moreover, our findings suggest a minor exocyst role in hemicellulose remodeling, consistent with studies linking exocyst to unconventional secretion of xyloglucan-related enzymes (De Caroli et al., 2021). The guard cells’ pectin methylation states are in agreement with results published by Amsbury et al. (2016), where the less de-methylated stomata of the *pme6* mutant have problems in opening and closing dynamics due to altered cell wall stiffness. Interestingly, despite the strongest aperture deviation found for *exo70B2*, it is *exo70B1* that has the most altered pectin methylation, as observed by enhanced COS-Alexa488 staining of cotyledon stomatal cell walls. We speculate that in the case of *exo70B2*, the combination of specific differentially expressed genes may create an additive effect resulting in the prominent stomatal phenotype in *exo70B2*.

Our transcriptomic data further highlight potential candidate genes that may contribute to the “phenotypic rescue” observed in the *exo70B1×B2* double mutant. For instance, the expression of the XET gene AT5G57560 was strongly downregulated in *exo70B1* and moderately reduced in *exo70B2*, yet restored to near wild-type levels in the double mutant. Additional candidate genes that may influence the cross-talk between stomatal and cell wall functions are listed in **Tables 2**-**4**. However, the specific roles of these genes in potential mutational compensation remain to be experimentally validated.

The upstream triggers of altered cell wall architecture in *exo70B* mutants remain unclear. One plausible mechanism could be defective exocytosis of wall-modifying enzymes, including pectin and hemicellulose regulators, during developmental or immune responses. Although further physiological validation is necessary, our findings are compatible with the view that EXO70B isoforms may function as tethering factors in UPS, potentially involving multivesicular bodies/late endosomes (MVBs/LEs), analogous to the role of SYP121/PEN1, a SNARE protein involved in exosome-like secretion during immunity (Elias et al., 2003; Kulich et al., 2013; Nielsen and Thordal-Christensen, 2013; Ortmannová et al., 2022; Rutter and Innes, 2017). We hypothesize that EXO70Bs affect cell wall quality via specialized versions of the exocyst complex, with EXO70 subunit specifically interacting with distinct domains of the plasma membrane (Synek et al., 2021). However, EXO70Bs also influence the protein composition of the plasma membrane, as evidenced by the altered trafficking of the FLS2 receptor in *exo70B* mutants (Wang et al., 2020). Consistent with this, our RNA-seq data revealed an upregulation of the S-type anion channel, SLAC1, in *exo70B* mutants following chitosan treatment. This finding suggests that the EXO70Bs are required for the proper activation of the pathway leading to stomatal closure (Hedrich and Geiger, 2017; Koers et al., 2011).

Importantly, a disruption in cell wall modifications is known to activate defense signaling via cell wall integrity (CWI) surveillance mechanisms, which detect perturbations and initiate damage-associated molecular pattern (DAMP)-triggered immunity (DTI) (Bacete et al., 2018; Wolf et al., 2012). Furthermore, various metabolites released during cell wall remodeling may perform priming effects leading to enhanced defense readiness (Hann et al., 2014; Swaminathan et al., 2021). In the case of *exo70B* mutants, we propose that defense-related gene expression activation may be a consequence of such priming by disturbed cell wall integrity, leading to signals that influence chromatin remodeling. In *exo70B2*, the previously reported interaction with MAPK3 suggests a potential role for MAPK signaling in this process (Brillada et al., 2021), which is also reflected in the slight upregulation of MAPK3 expression in *exo70B2*. Nevertheless, our MAPK kinase assay excludes the hyperactivation via the MAP kinase cascade, suggestive of other signaling pathways involved, a possibility that remains to be further inspected (Harris et al., 2023). Moreover, in the case of *exo70B1*, the primed state, together with the absence of EXO70B1 interactions with regulatory proteins such as TN2 and RIN4 in the LOF mutant, may result in the excessive SA biosynthesis and activation of HR-type immunity (Sabol et al., 2017; Zhao et al., 2015).

Altogether, our findings indicate that EXO70B1 and EXO70B2 are involved in multifaceted and often distinct aspects of stomatal regulation, cell wall remodeling, and immune priming—three interrelated functions that can also be independently modulated. In our effort to connect secretion- and defense-related modifications of the cell wall (including those in guard cells) with the transcriptional phenotypes observed in the single and double mutants, we uncovered that the interplay between EXO70B1 and EXO70B2 is complex—sometimes synergistic, at other times divergent—potentially reflecting their relatively recent duplication within the Brassicaceae. Future work should aim to dissect the cell type-specific functions of these isoforms, define their cargo selectivity, and clarify their respective contributions to conventional and unconventional secretion. Such insights will be critical for understanding how plants balance growth and defense through fine-tuned exocyst specialization.

## Data Availability

The data presented in the study are deposited in the GEO repository, under the accession numbers GSE311031 and GSE311032.

## Supplementary material

Supplementary Table 1 A basic information on the RNA-seq quality information, including the type of sequencing, sequencing depth, and the number of biological replicates.

Supplementary Table 2 A list of significantly up- and down-regulated genes, and significant gene enrichment within GO categories for *exo70B1*, *B2* and double mutant *B1xB2* lines in comparison to WT, in seedling stage.

Supplementary Table 3 A list of significantly up- and down-regulated genes, and significant gene enrichment within GO categories for *exo70B1*, *B2* and double mutant *B1xB2* lines in comparison to WT, in the adult stage, mock and chitosan treated.

Supplementary Table 4 **T**he overlap of genes involved in response to abscisic acid (GO:0009738), with significantly differentially expressed genes in *exo70B* mutants.
