# Supplementary material for: Exocyst subunits EXO70B1 and B2 contribute to stomatal dynamics and cell wall modifications
Source: Front Plant Sci. 2025 Dec 17;16:1694769. doi: 10.3389/fpls.2025.1694769 (PMC12753983; doi:10.3389/fpls.2025.1694769)
Supplement: Supplementary file 5 [file DataSheet1.pdf]

A

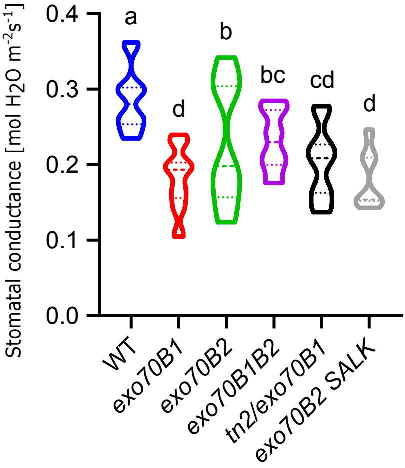

B

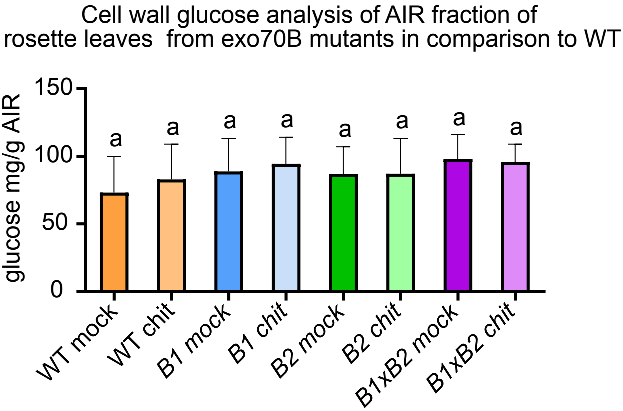

Supplementary Figure 1. A) Plot shows steady-state (400 ppm) stomatal conductance level of WT and mutants including second mutant version *exo70B2* Salk line and *exo70B1/tn2* double mutant. One-way ANOVA with post-hoc Tukey HSD was used for statistics, $p< 0.05$, $n> 5$ independent leaves per genotype, error bars represent SD. B) Cell wall analysis of mock and chitosan treated *exo70B* mutants in comparison to WT. Cell wall glucose amounts in *exo70B* mutants' cell walls are at the similar level as in WT plants; n = 8-9; two-way ANOVA $p< 0.05$.
