# Supplementary material for: Exocyst subunits EXO70B1 and B2 contribute to stomatal dynamics and cell wall modifications
Source: Front Plant Sci. 2025 Dec 17;16:1694769. doi: 10.3389/fpls.2025.1694769 (PMC12753983; doi:10.3389/fpls.2025.1694769)
Supplement: Supplementary file 6 [file DataSheet2.pdf]

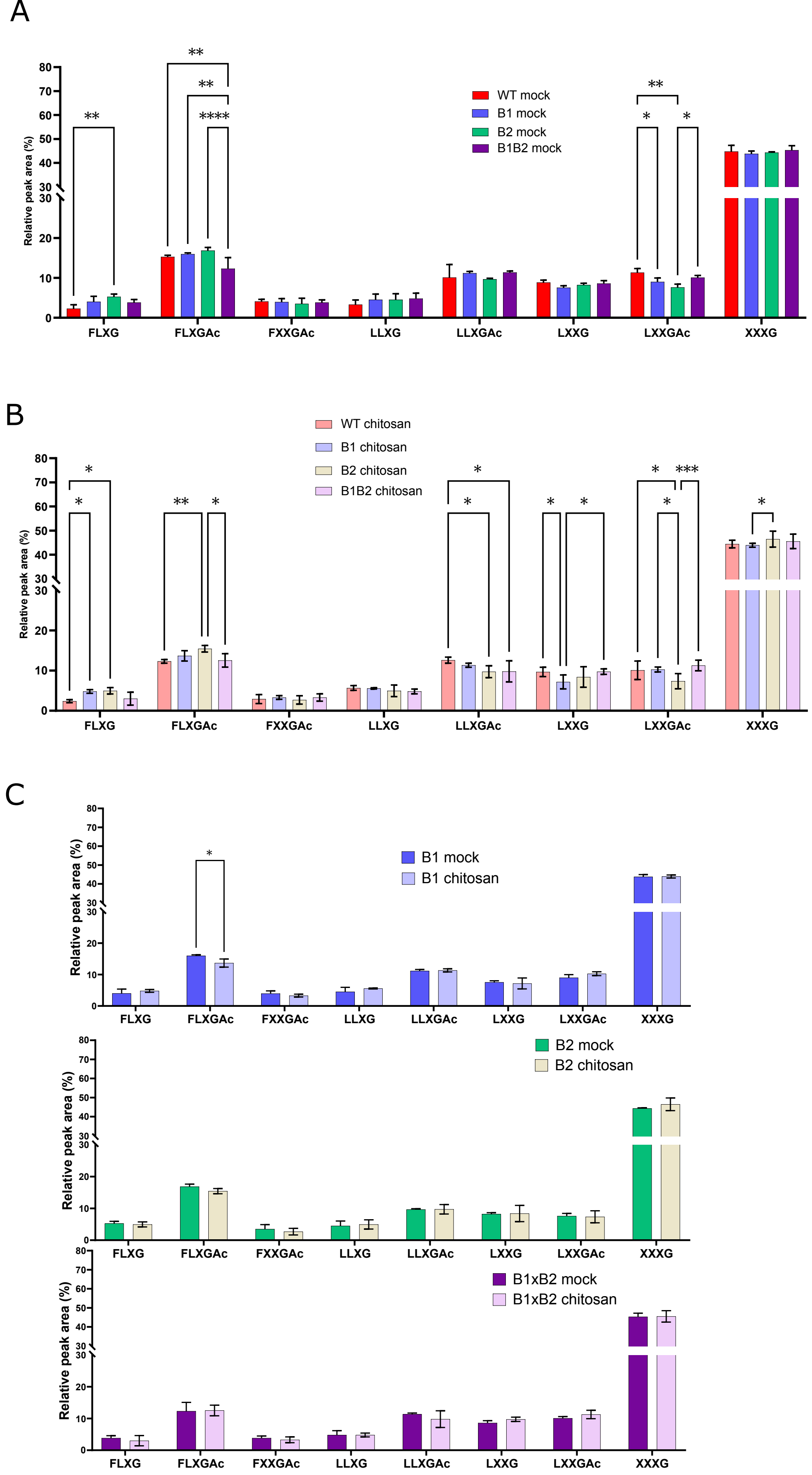

Supplementary Figure 2. A) Enzymatic fingerprinting of hemicellulose upon the mock treatment; the mutant *exo70B* lines have slightly more of acetylated hemicellulose. B) Enzymatic fingerprinting of hemicellulose upon the chitosan treatment; similarly to mock treatment, the *exo70B* mutant lines have more of acetylated hemicellulose. C) Comparison of hemicellulose composition of mock- and chitosan-treated plants for each line. Oligosaccharide nomenclature follows (Fry et al., 1993): G - glucose; X - xylose-glucose; L -galactose-xylose-glucose; F -fucose-galactose-xylose-glucose; Y - galacturonic acid-xylose-glucose; Ac: acetyl ester group. Data represents mean ±SD, n = 4, $*p < 0.05$, $**p < 0.01$, student's t-test.
