# Supplementary material for: Exocyst subunits EXO70B1 and B2 contribute to stomatal dynamics and cell wall modifications
Source: Front Plant Sci. 2025 Dec 17;16:1694769. doi: 10.3389/fpls.2025.1694769 (PMC12753983; doi:10.3389/fpls.2025.1694769)
Supplement: Supplementary file 8 [file DataSheet4.pdf]

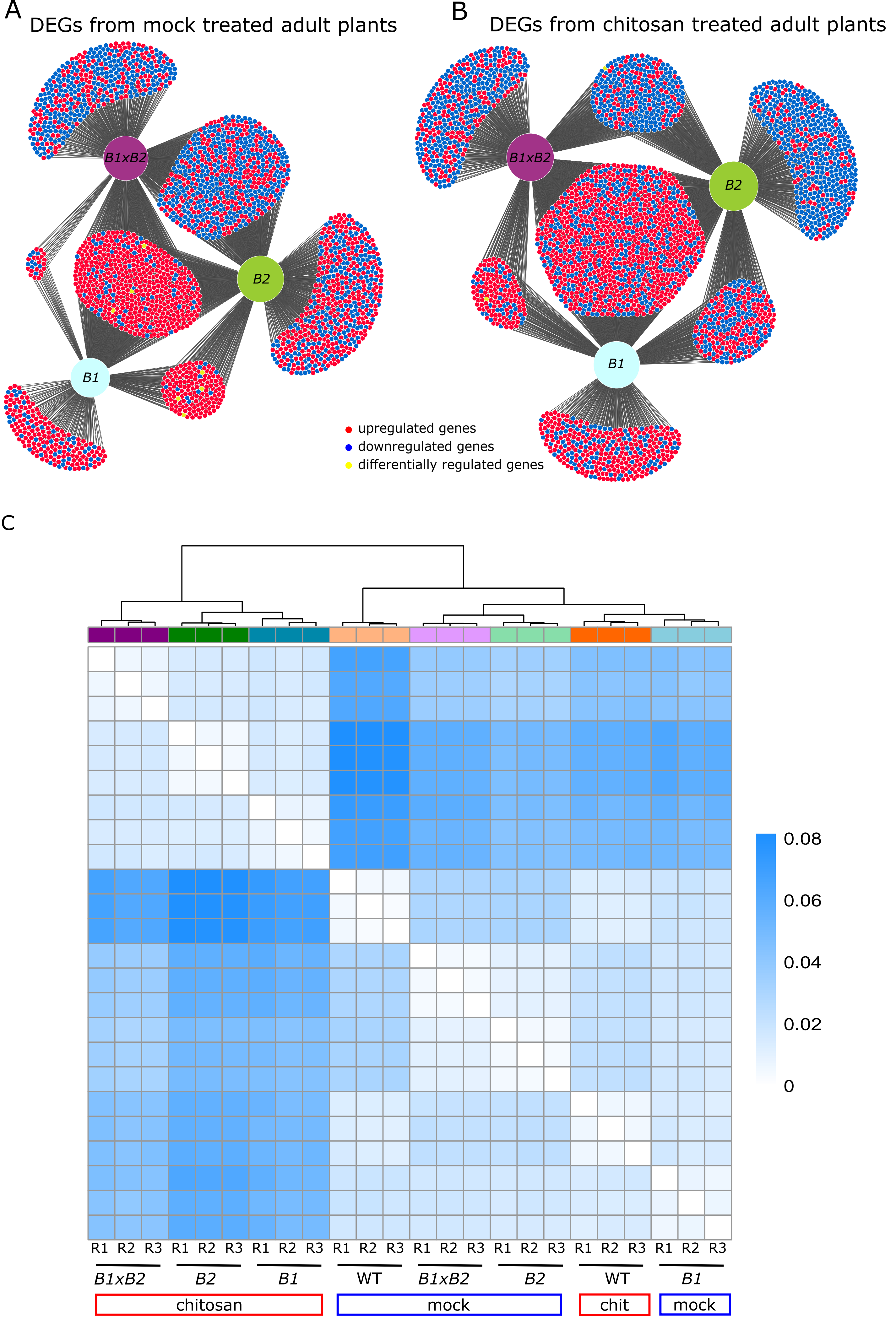

Supplementary Figure 4. General features of RNAseq analysis of *exo70Bs* adult plants. A) DiVenn diagram demonstrates similarities and differences in sets of DEGs determined for *B1* vs. WT, *B2* vs. WT and *B1xB2* vs. WT RNAseq comparisons. B) DiVenn diagrams demonstrate similarities and differences in sets of DEGs determined for chitosan treated WT and each of *exo70B* mutant lines upon chitosan treatment. C) Quality control of quantification of transcriptomic profiling of adult plants, mock (underlined with blue box) and chitosan treated (underlined with red box), from wild-type and genotypes *exo70B1* (*B1*), *exo70B2* (*B2*) and double mutant *exo70B1xB2* (*B1xB2*). Heatmap shows Jensen-Shannon divergence coefficients for individual sample comparisons.ermined for *B1* vs. WT, *B2* vs. WT and *B1xB2* vs. WT RNAseq comparisons. hannon divergence coefficients for individual sample comparisons.
