# Supplementary material for: Exocyst subunits EXO70B1 and B2 contribute to stomatal dynamics and cell wall modifications
Source: Front Plant Sci. 2025 Dec 17;16:1694769. doi: 10.3389/fpls.2025.1694769 (PMC12753983; doi:10.3389/fpls.2025.1694769)
Supplement: Supplementary file 9 [file DataSheet5.pdf]

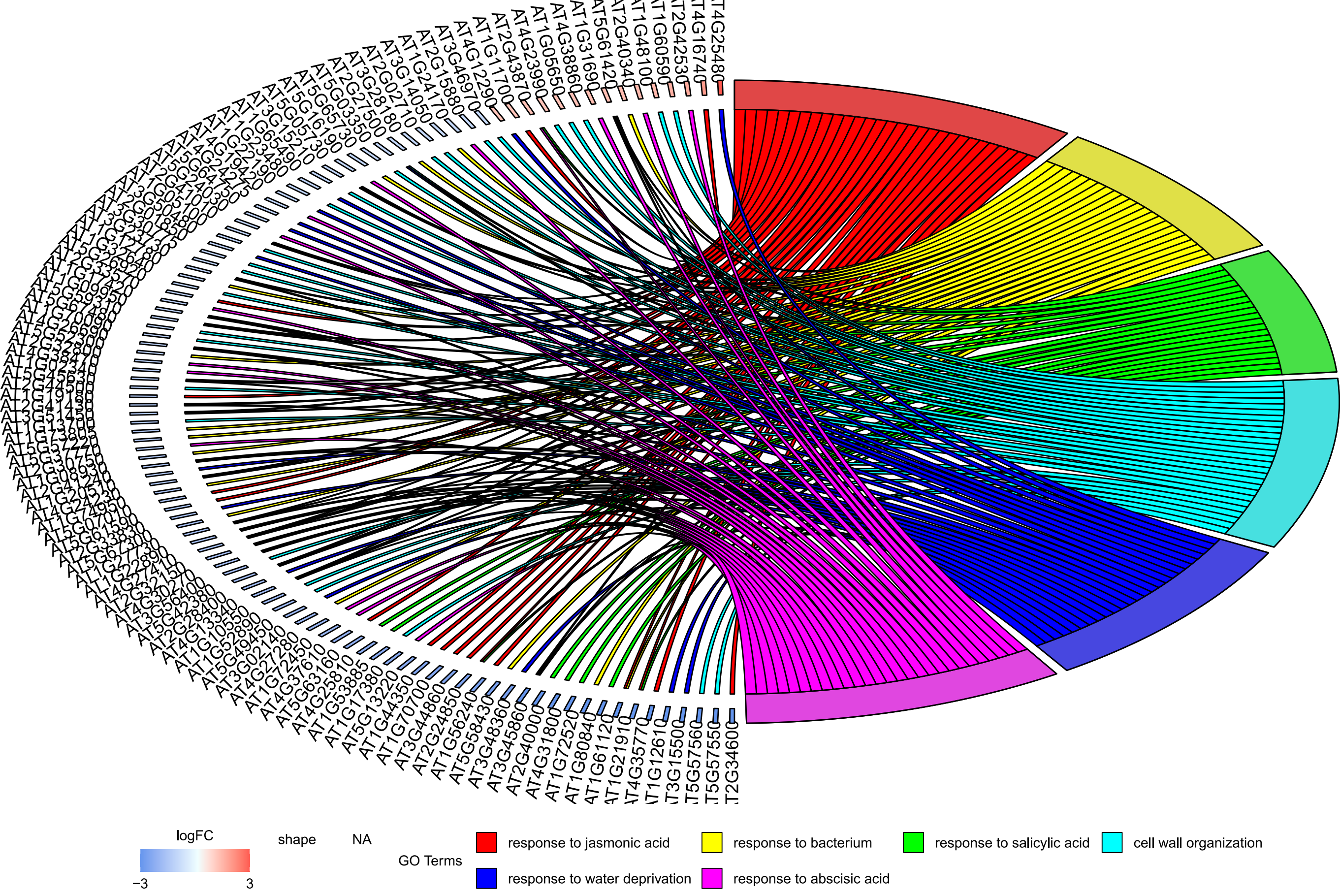

Supplementary Figure 5. Chord diagram showing the differentially expressed genes of *exo70B1* seedlings (compared to WT control) found to associate with several selected GO categories (on the right). The color code of fold change of genes' expression is shown on the left.

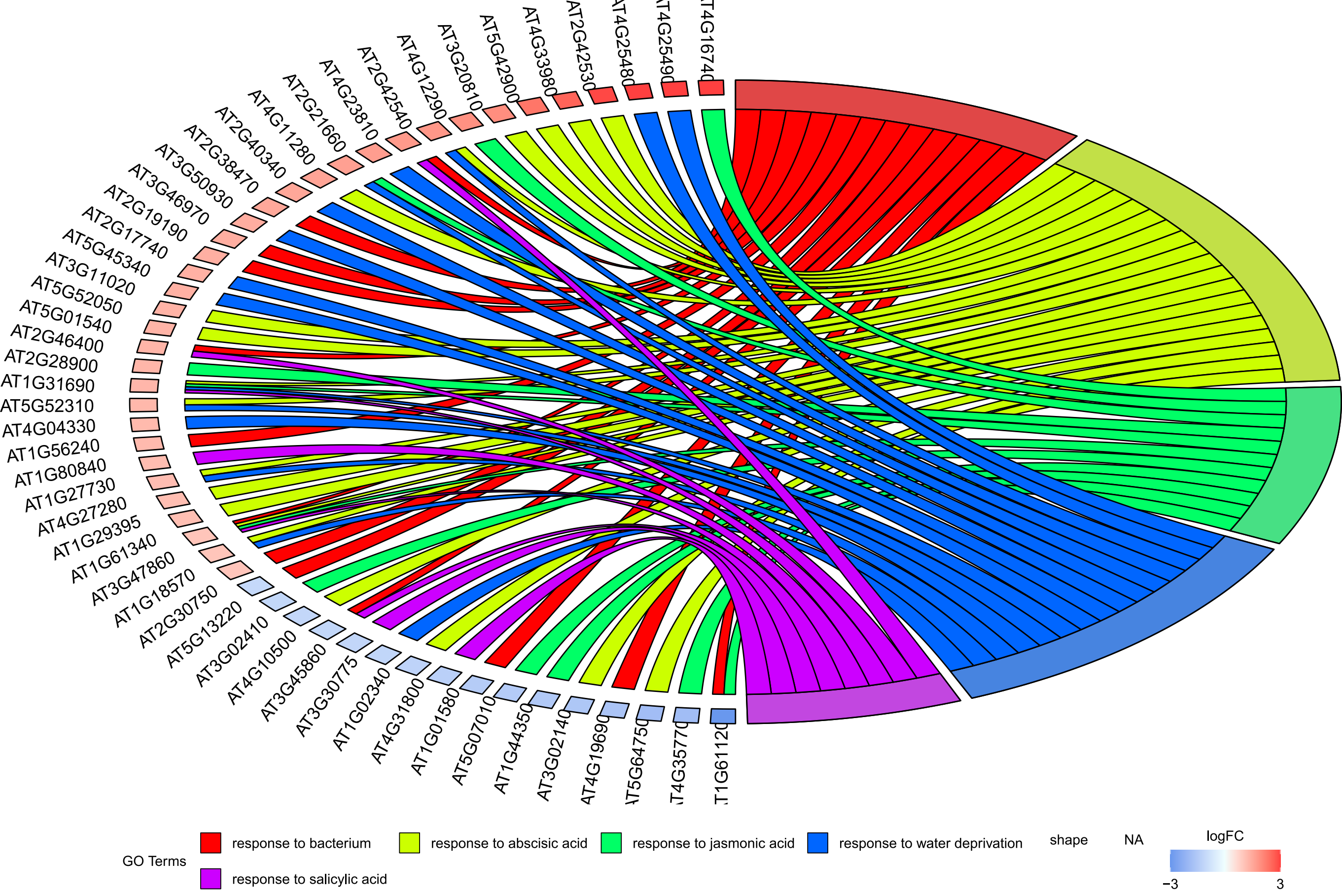

Supplementary Figure 6. Chord diagram showing the DEGs of *exo70B2* seedlings (compared to WT control) found to associate with several selected GO categories pathways (on the right). The color code of the fold change of genes' expression is shown on the right.

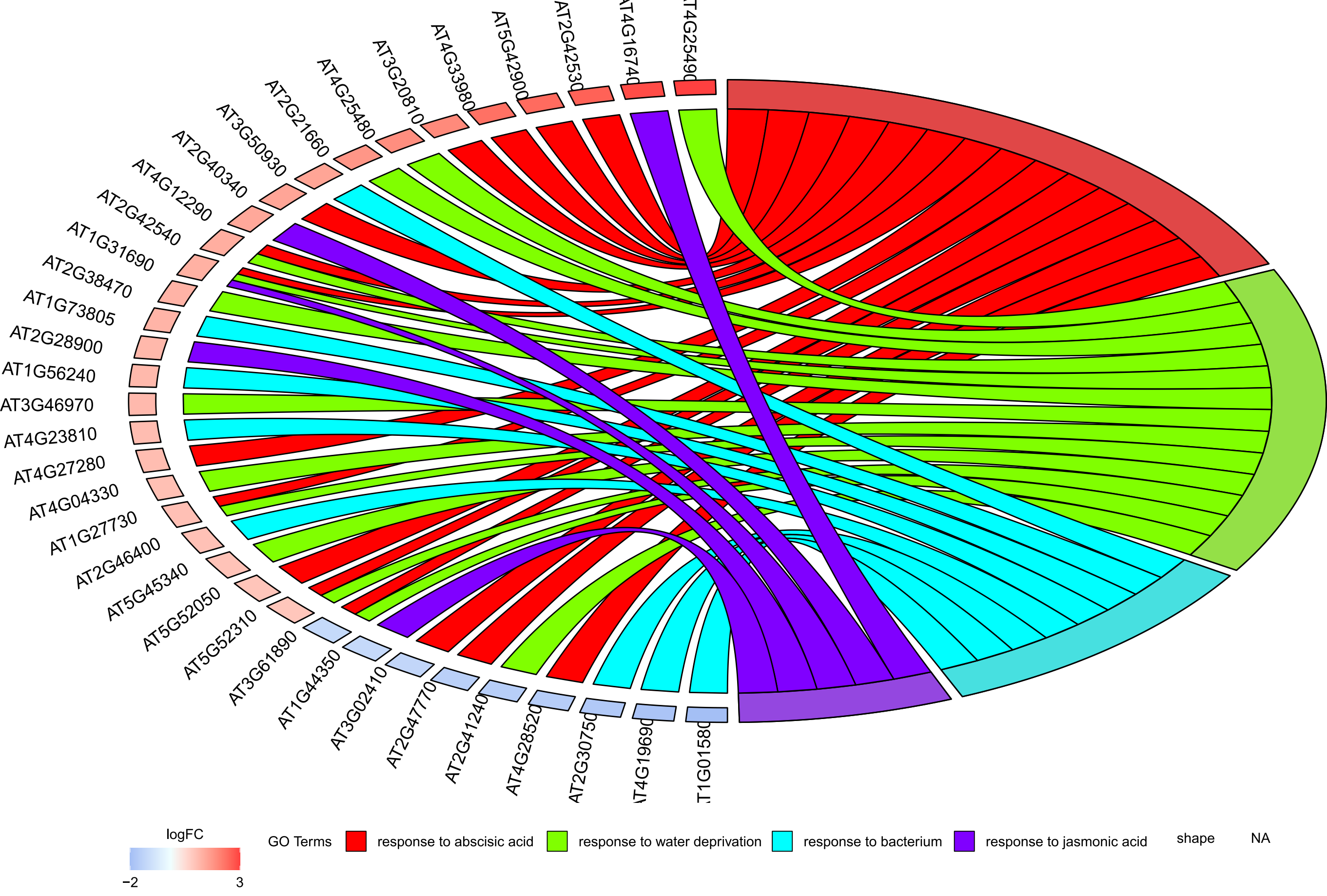

Supplementary Figure 7. Chord diagram showing the DEGs of *exo70B1xB2* seedlings (compared to WT control) found to associate with several selected GO categories (on the right). The color code of the fold change of genes' expression is shown on the left.
