# Supplementary material for: Exocyst subunits EXO70B1 and B2 contribute to stomatal dynamics and cell wall modifications
Source: Front Plant Sci. 2025 Dec 17;16:1694769. doi: 10.3389/fpls.2025.1694769 (PMC12753983; doi:10.3389/fpls.2025.1694769)
Supplement: Supplementary file 10 [file DataSheet6.pdf]

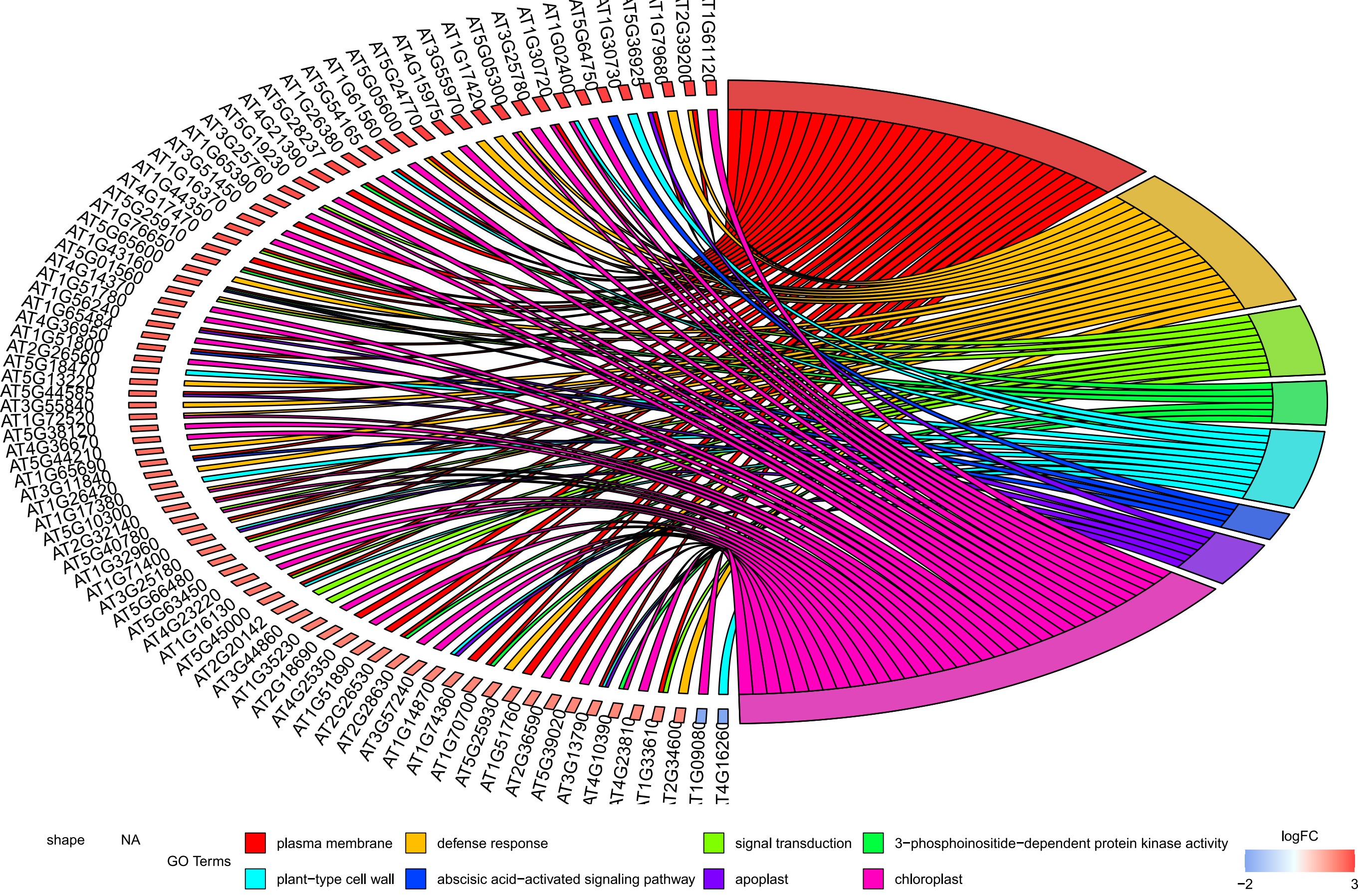


**Supplementary Figure 8.** Chord diagram showing the DEGs of *exo70B1* adult plants (compared to WT control) found to associate with several selected GO categories (on the right). The color code of the fold of genes' expression is shown on the right.

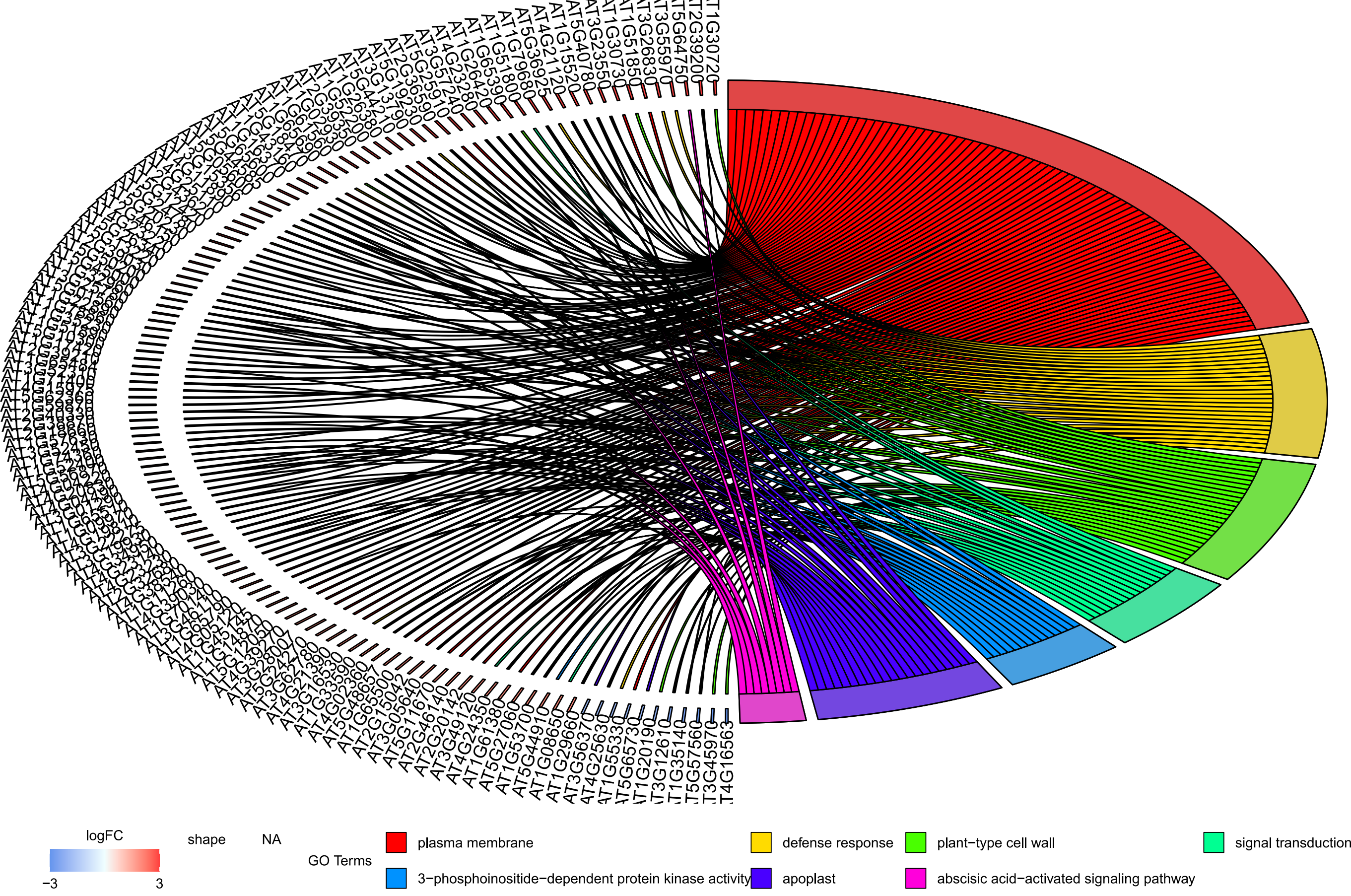


**Supplementary Figure 9.** Chord diagram showing the DEGs of *exo70B2* adult plants (compared to WT control) found to associate with several selected GO categories (on the right). The color code of the fold change of core genes is shown on the left.

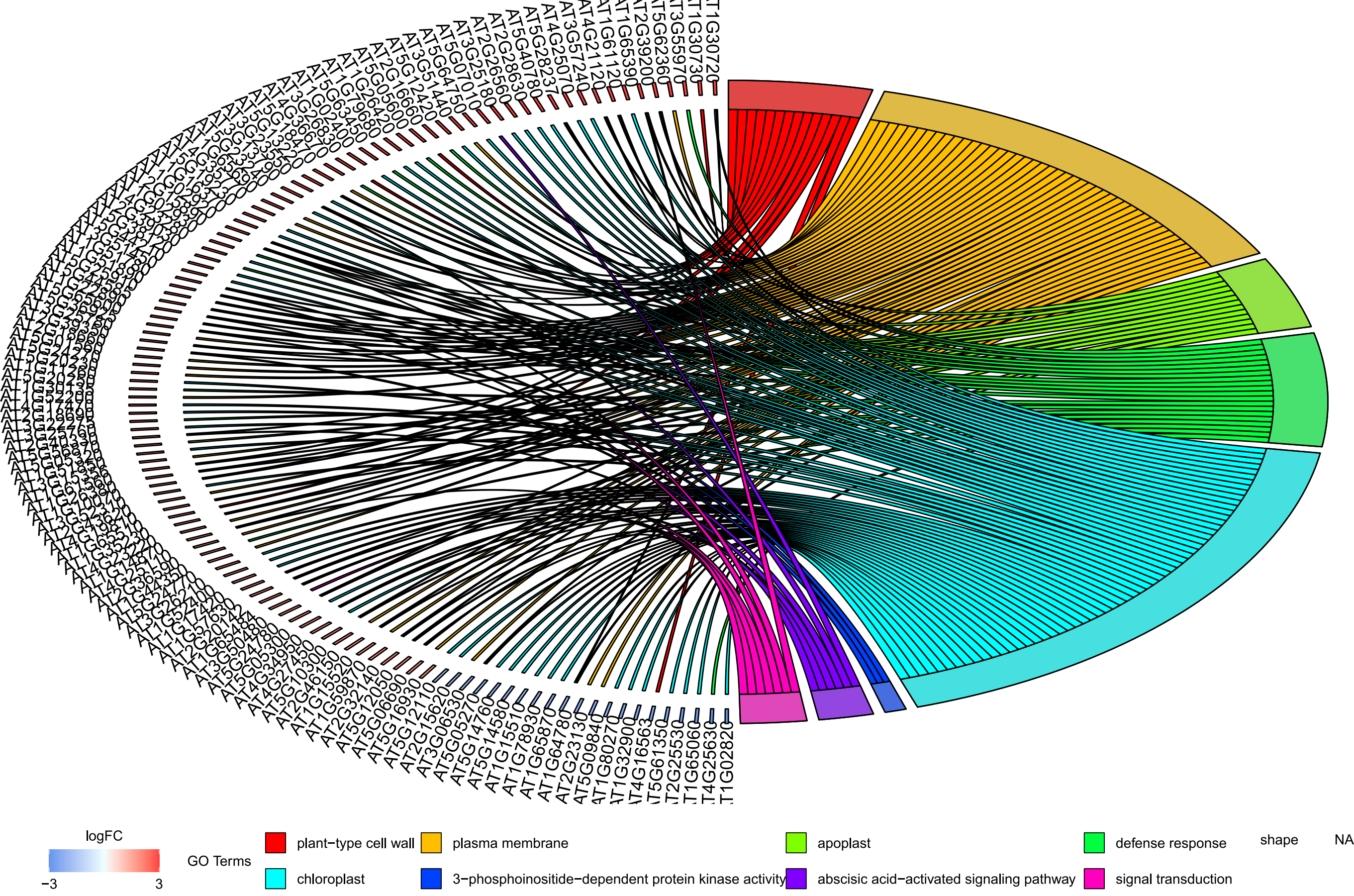


**Supplementary Figure 10.** Chord diagram showing the DEGs of *exo70B1xB2* adult plants (compared to WT control) found to associate with several selected GO categories (on the right). The color code of the fold change of core genes is shown on the left.
