# Supplementary material for: Exocyst subunits EXO70B1 and B2 contribute to stomatal dynamics and cell wall modifications
Source: Front Plant Sci. 2025 Dec 17;16:1694769. doi: 10.3389/fpls.2025.1694769 (PMC12753983; doi:10.3389/fpls.2025.1694769)
Supplement: Supplementary file 11 [file DataSheet7.pdf]

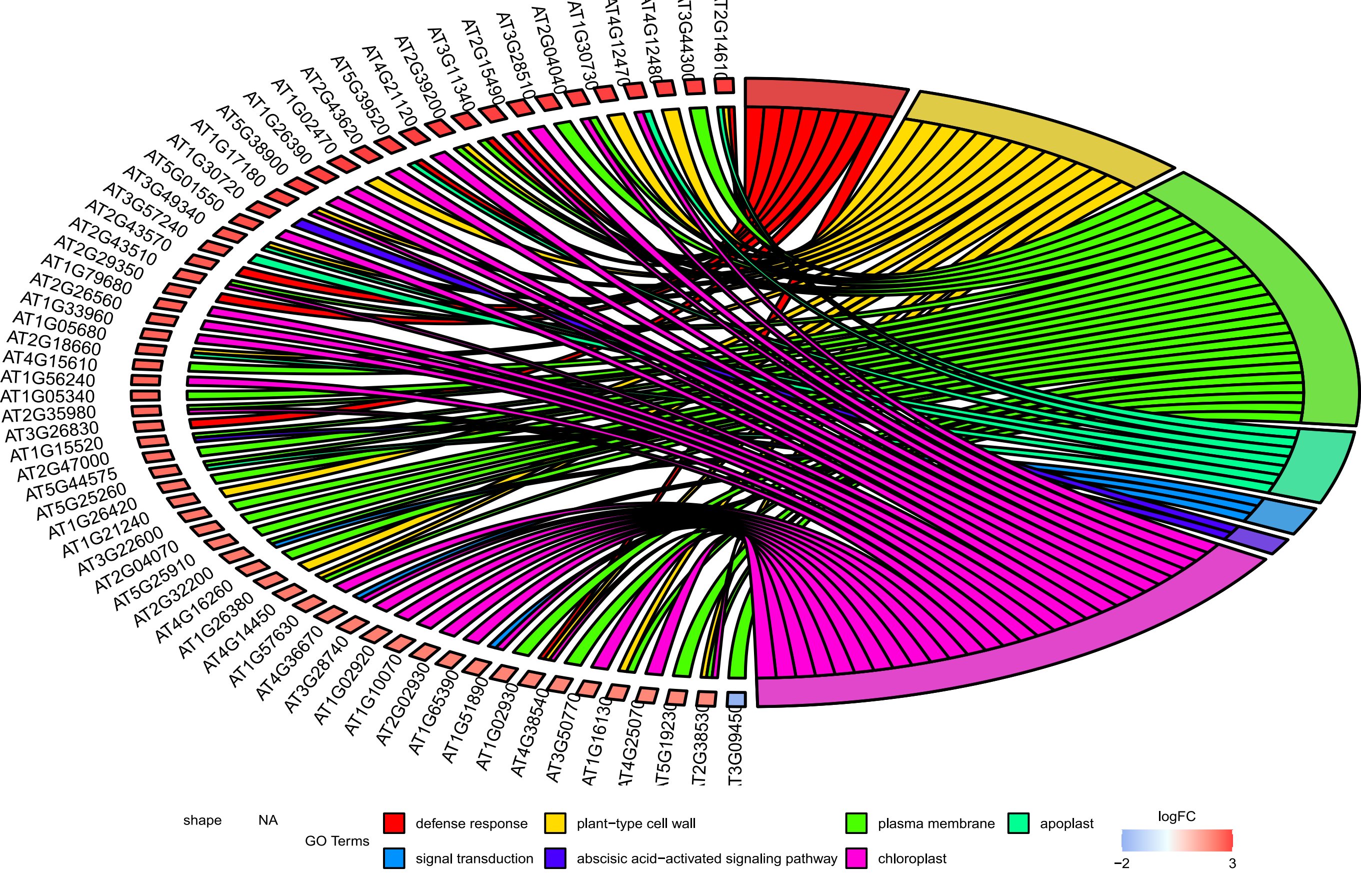

Supplementary Figure 11. Chord diagram showing the DEGs found to associate with several selected GO categories (on the right) for chitosan-treated WT adult plants, in comparison to mock treated WT. The color code of the fold change of genes' expression is shown on the right.

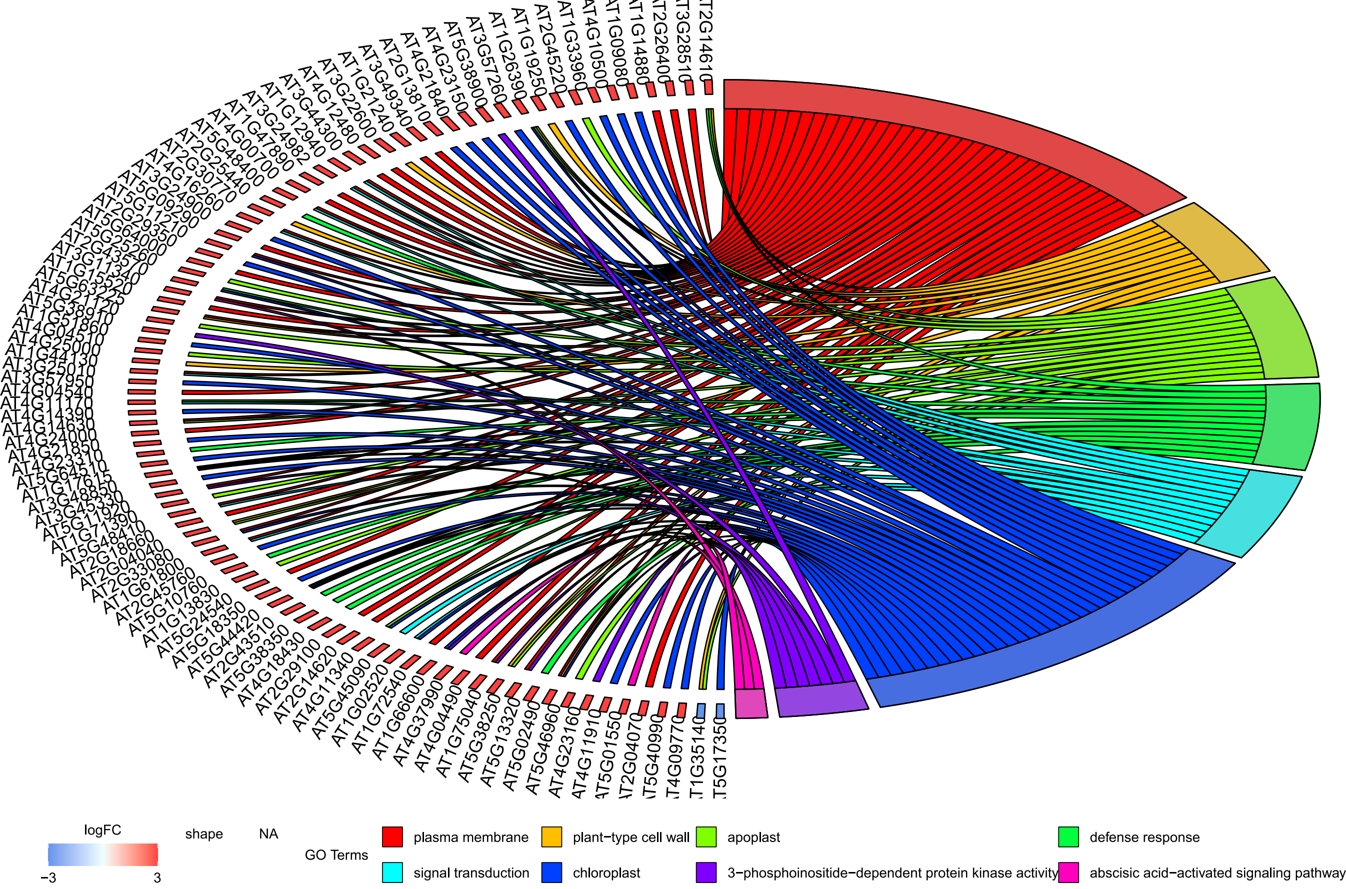

Supplementary Figure 12. Chord diagram showing the DEGs found to associate with several selected GO categories (on the right) for chitosan-treated *exo70B1* adult plants, in comparison to mock treated *exo70B1*. The color code of the fold change of genes' expression is shown on the left.

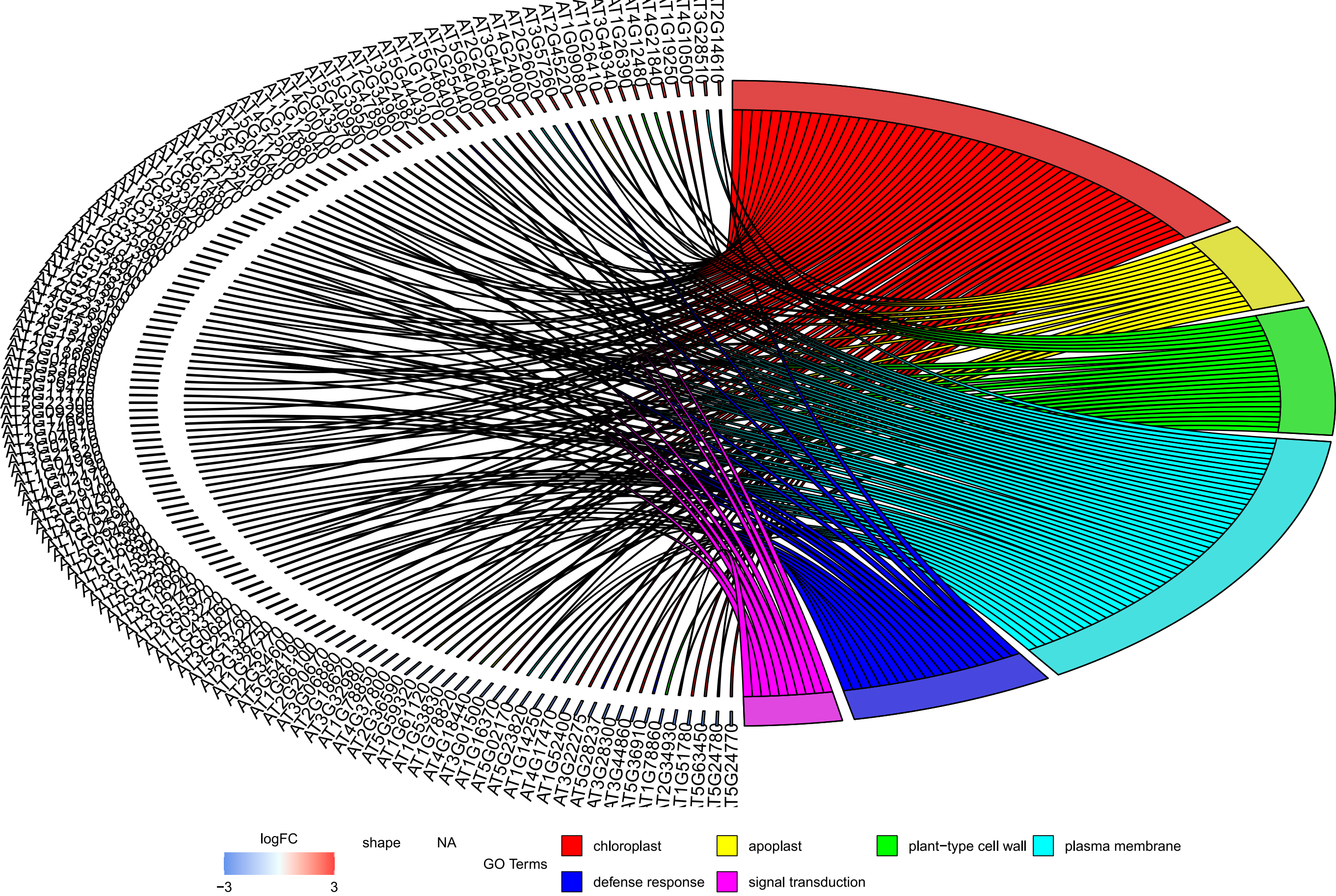

Supplementary Figure 13. Chord diagram showing the DEGs found to associate with several selected GO categories (on the right) for chitosan-treated *exo70B2* adult plants, in comparison to mock treated *exo70B2*. The color code of the fold change of genes' expression is shown on the left.

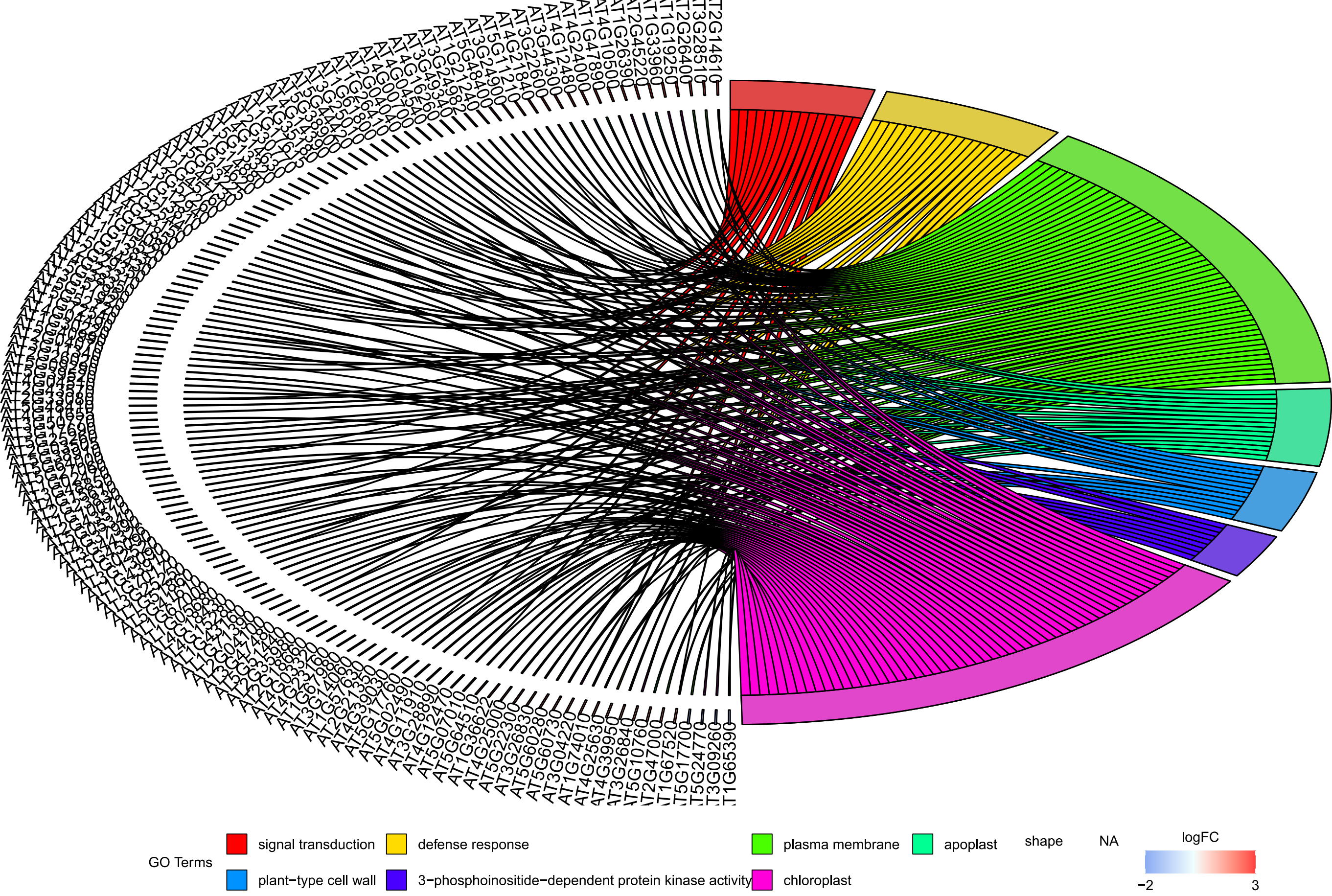

Supplementary Figure 14. Chord diagram showing the DEGs found to associate with several selected GO categories (on the right) for chitosan-treated *exo70B1xB2* adult plants, in comparison to mock treated *exo70B1xB2*. The color code of the fold change of genes' expression is shown on the right.
