# Supplementary material for: Exocyst subunits EXO70B1 and B2 contribute to stomatal dynamics and cell wall modifications
Source: Front Plant Sci. 2025 Dec 17;16:1694769. doi: 10.3389/fpls.2025.1694769 (PMC12753983; doi:10.3389/fpls.2025.1694769)
Supplement: Supplementary file 12 [file DataSheet8.pdf]

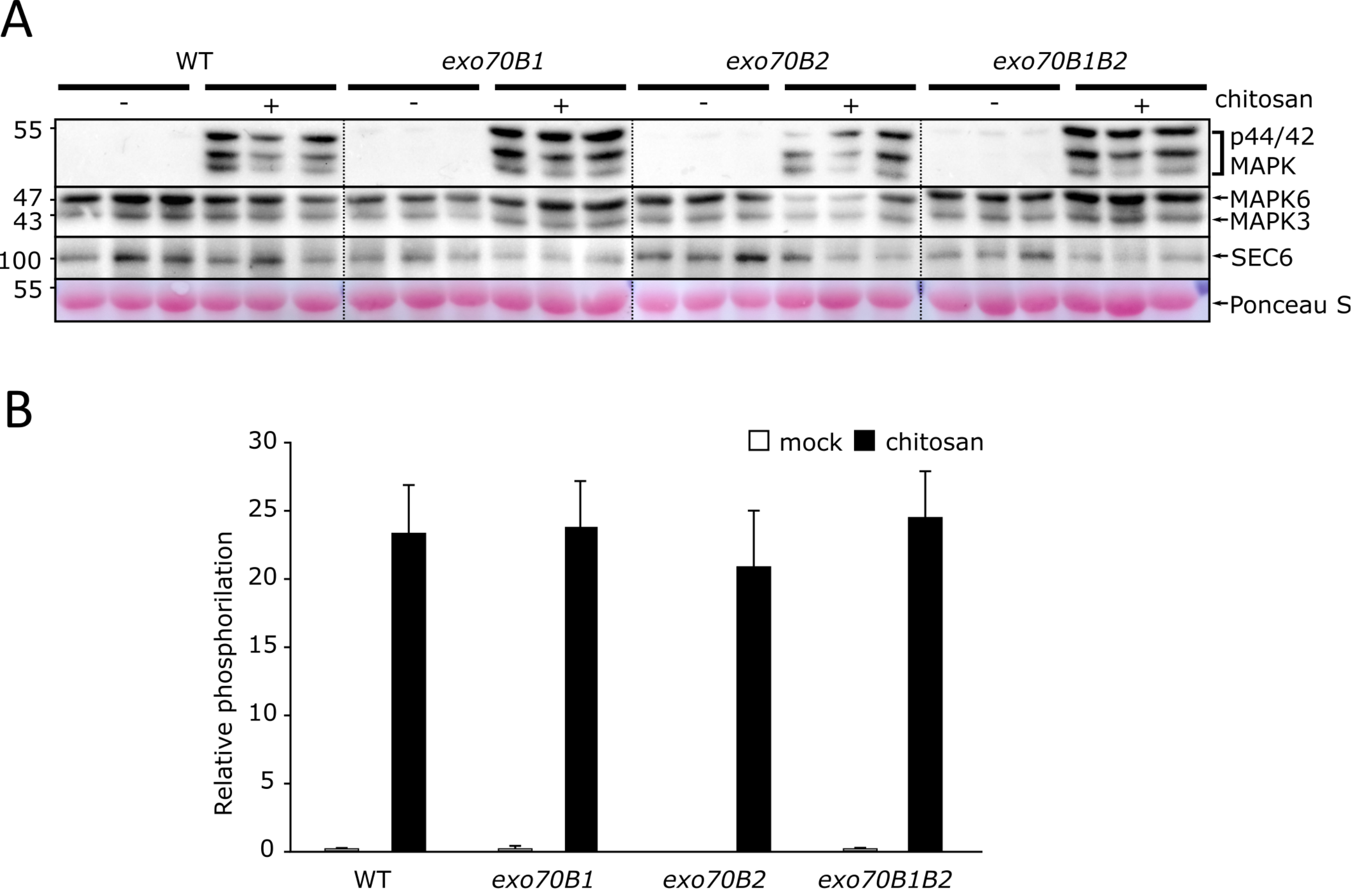

Supplementary Figure 15. MAPK activation assays in exo70B mutants. A) Western blot analysis of MAPK activation by phosphorylation upon 30 min of chitosan treatments in seedlings of *exo70B1*, *B2* and double *B1xB2* mutants, always as three replicates R1-3. B) Relative MAPKs phosphorylation quantified as the ratio of band intensities obtained for phosphorylated portions versus the whole pool MAPKs. The chitosan treatment in all analyzed lines causes significant increase in MPK3/6/ phosphorylation in comparison to the mock treatment. No significant difference among lines according to ANOVA test has been found neither for mock nor for chitosan treated plants.
